# Geographic mosaic of symbiont selectivity in a genus of epiphytic cyanolichens

**DOI:** 10.1002/ece3.343

**Published:** 2012-08-10

**Authors:** Katja Fedrowitz, Ulla Kaasalainen, Jouko Rikkinen

**Affiliations:** 1 Department of Ecology, Swedish University of Agricultural Sciences (SLU)P.O. 7044, SE-750 07, Uppsala, Sweden; 2 Department of Biosciences, University of HelsinkiP.O. 65, 00014, Helsinki, Finland

**Keywords:** Cyanobacteria, functional guild, *Nephroma*, *Nostoc*, symbiosis

## Abstract

In symbiotic systems, patterns of symbiont diversity and selectivity are crucial for the understanding of fundamental ecological processes such as dispersal and establishment. The lichen genus *Nephroma* (Peltigerales, Ascomycota) has a nearly cosmopolitan distribution and is thus an attractive model for the study of symbiotic interactions over a wide range of spatial scales. In this study, we analyze the genetic diversity of *Nephroma* mycobionts and their associated *Nostoc* photobionts within a global framework. The study is based on Internal Transcribed Spacer (ITS) sequences of fungal symbionts and tRNA^L^^eu^ (UAA) intron sequences of cyanobacterial symbionts. The full data set includes 271 *Nephroma* and 358 *Nostoc* sequences, with over 150 sequence pairs known to originate from the same lichen thalli. Our results show that all bipartite *Nephroma* species associate with one group of *Nostoc* different from *Nostoc* typically found in tripartite *Nephroma* species. This conserved association appears to have been inherited from the common ancestor of all extant species. While specific associations between some symbiont genotypes can be observed over vast distances, both symbionts tend to show genetic differentiation over wide geographic scales. Most bipartite *Nephroma* species share their *Nostoc* symbionts with one or more other fungal taxa, and no fungal species associates solely with a single *Nostoc* genotype, supporting the concept of functional lichen guilds. Symbiont selectivity patterns within these lichens are best described as a geographic mosaic, with higher selectivity locally than globally. This may reflect specific habitat preferences of particular symbiont combinations, but also the influence of founder effects.

## Introduction

In a lichen symbiosis, the heterotrophic fungal partner (mycobiont) requires a photoautotrophic symbiont (photobiont) to obtain fixed carbon and it consequently needs to recruit suitable photobionts from its surroundings if it disperses solely by fungal spores. However, it is still largely unknown how lichen mycobionts find and select their photobionts, and how well the compatible symbionts identified by lichen mycobionts correspond to the photobiont genotypes that we can presently identify with molecular markers.

In lichens, symbiont selectivity has usually been studied from the fungal perspective as fungal symbionts seem to often be more selective toward their photobionts than vice versa (Tschermak-Woess 1988; Rikkinen 1995, 2003; Piercey-Normore and DePriest 2001; Elvebakk et al. 2008; Otálora et al. 2010). Photobiont selectivity can be understood as the preferred association of a specific lichen mycobiont with particular photobiont genotypes. Patterns of selectivity have been studied in different groups of lichens, including many cyanolichens that have cyanobacteria of the genus *Nostoc* as photobionts. Different degrees of selectivity have been reported, ranging from low to very high (e.g., Paulsrud et al. 1998, 2000, 2001; Rikkinen et al. 2002; Wirtz et al. 2003; O'Brien et al. 2005; Stenroos et al. 2006; Myllys et al. 2007; Elvebakk et al. 2008; Otálora et al. 2010; Fedrowitz et al. 2011).

Patterns of symbiont diversity in lichens may be influenced by many interacting factors, including symbiont availability, reproductive strategy, the abiotic environment, and the specific ecological requirements of each symbiont (e.g., Beck et al. 2002; Rikkinen 2003; Blaha et al. 2006; Yahr et al. 2006; Muggia et al. 2008; Lücking et al. 2009; Otálora et al. 2010; Fernández-Mendoza et al. 2011; Peksa and Škaloud 2011; Piercey-Normore and Deduke 2011; Kaasalainen et al. 2012). In cyanolichens, sequence identical *Nostoc* tRNA^Leu^ (UAA) genotypes have been found both locally (Paulsrud et al. 1998; Fedrowitz et al. 2011) and from different regions (Paulsrud et al. 2000; Rikkinen 2004; Summerfield and Eaton-Rye 2006), but geographic patterns of symbiont selection have rarely been studied. However, global approaches to symbiont diversity are crucial for a better understanding of ecological processes affecting lichen-forming fungi, including their ability to select appropriate symbiotic partners and to colonize new habitats.

**Figure 1:**
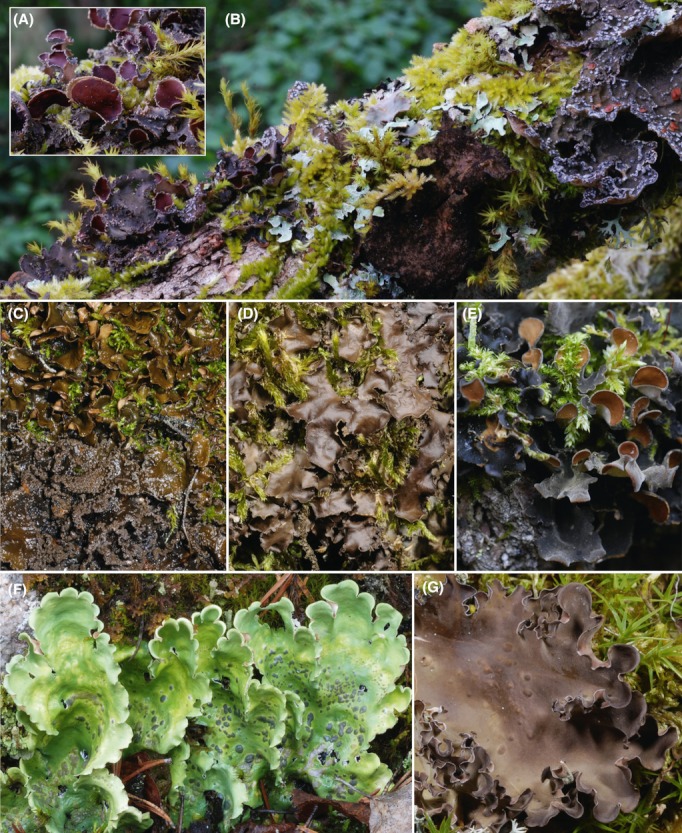
*Nephroma* species. (A) Close-up of *Nephroma helveticum* from Oregon. (B) Overview of the same lichen in its typical habitat together with two other *Nephroma*-guild cyanolichens: *Sticta fuliginosa* in the middle and *Pseudocyphellaria anomala* on the right. (C) *Nephroma bellum* (upper lichen) and *N. parile* (lower lichen) on moist boulder in Finnish Lapland. (D) *Nephroma laevigatum* on tree trunk from southern Spain. (E)*Nephroma resupinatum* on tree trunk from central Finland. (F) *Nephroma arcticum* on moist rock face in Finnish Lapland. (G) *Nephroma expallidum* on subarctic tundra soil in Finnish Lapland. [Correction added on 15 August 2012, after first online publication: The species name in Fig. 1E has been changed from Nephroma laevigatum to Nephroma resupinatum].

The genus *Nephroma* (Peltigerales, Ascomycota) includes about 40 species of foliose macrolichens (James and White 1987; White and James 1988; Lohtander et al. 2002; Sérusiaux et al. 2011). They all have a stratified thallus with a well-developed cortex on both surfaces (Fig. 1). Apothecia develop on the lower surface of thallus lobes, which are later reflexed to expose the hymenium (Fig. 1A, B and E). In addition to fungal ascospores, most species also produce symbiotic propagules (soredia, isidia, or lobules), in which the mycobiont and photobiont are dispersed together (Fig. 1C). Most *Nephroma* species are bipartite lichens and house cyanobacteria of the genus *Nostoc* as photobionts (Fig. 1A–E). Fewer species (e.g., *Nephroma arcticum*) are tripartite lichens (Fig. 1F–G), consisting of a mycobiont in association with both a photosynthetic green alga (*Coccomyxa*) and a nitrogen-fixing cyanobacterium (*Nostoc*). In tripartite species, the cyanobacterial symbiont is confined to internal cephalodia in the medulla (Fig. 1F) or external cephalodia on the lower surface (Fig. 1G). Most bipartite *Nephroma* species are epiphytic and/or lithophytic and commonly occur in moist old-growth forests and similar humid habitats. All tripartite species in the Northern Hemisphere are terricolous and commonly grow among ground layer bryophytes in subarctic forests and peatlands. Some tripartite *Nephroma* species can also produce photosymbiodemes, that is, pairs of disparate morphs originating from symbiosis of the same fungus with two different photobiont types. The morphotypes may occur as independent or joined thalli and in the latter case the green algal morph seems to always originate from a primary cyanobacterial morph (Tønsberg and Holtan-Hartwig 1983; White and James 1988; Piercey-Normore et al. 2006). Several species of *Nephroma* are widely distributed on the Northern Hemisphere, and the genus, as a whole, has a nearly cosmopolitan distribution. This makes *Nephroma* an attractive model genus for the study of symbiotic association patterns over a wide range of geographic scales. Nevertheless, many currently accepted *Nephroma* species are quite variable both in thallus morphology and secondary chemistry, and are thought to include several chemical races (e.g., James and White 1987; White and James 1988; Vitikainen 2007) or undescribed species (e.g., Lohtander et al. 2002; Sérusiaux et al. 2011). Relevant information on the molecular phylogeny of the genus has already been gained in several studies (Lohtander et al. 2002, 2003; Piercey-Normore et al. 2006; Sérusiaux et al. 2011), and some data on the diversity of *Nostoc* photobionts have been obtained from studies on photobiont diversity patterns in epiphytic cyanolichen communities (e.g., Paulsrud et al. 1998, 2000; Rikkinen et al. 2002; Myllys et al. 2007; Fedrowitz et al. 2011). The latter studies have demonstrated that individual *Nephroma* species can house cyanobacterial symbionts identical to those of other cyanolichen species in the same environment, and thus engage in symbiotic interactions on the community scale (Rikkinen 2003).

This study aims to increase the general understanding of mycobiont–photobiont selectivity in lichen-forming fungi, with a focus on global geographic patterns and exemplified by the genus *Nephroma*. We pursue our goal by determining the genetic identities of *Nostoc* photobionts from a wide variety of *Nephroma* specimens from different parts of the world and superimposing the acquired information on a phylogenetic framework constructed for the fungal hosts. Emerging patterns of symbiotic selectivity are then discussed in the light of what is previously known about symbiont selectivity in *Nephroma* and lichens in general. Many epiphytic cyanolichens of the Peltigerales, including several bipartite *Nephroma* species, commonly occur in old-growth forests and are used as biological indicators of environmental continuity and for areas with high conservational value in temperate and boreal forests (Rose 1976, 1988; Kuusinen 1996; Nitare 2000; Coppins and Coppins 2002). Further studies into the basic biology of these symbioses are essential for the development of viable conservation strategies intended to maintain cyanolichen diversity in fragmenting forest ecosystems.

## Material and Methods

### Taxon sampling

**Figure 2:**
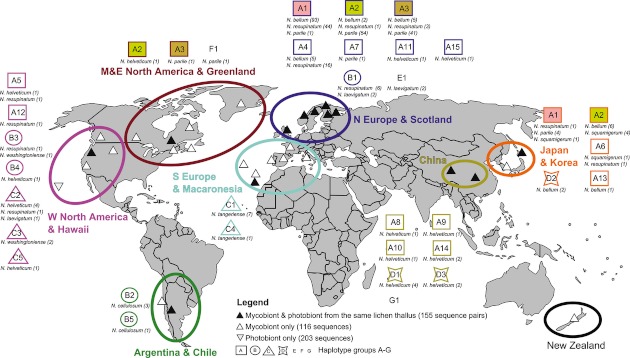
Geographic origin of sequences studied (small black and white triangles) and diversity of *Nostoc* photobionts on the basis of the tRNA^Leu^ (UAA) intron. The full data set included 271 mycobiont sequences and 358 photobiont sequences. Photobiont haplotype groups A–G are differentiated by assorted geometric forms with the respective genotypes denoted by numbers. Geographic regions are color coded. The respective lichen species and numbers of samples identified are shown below each *Nostoc* genotype. Sequence identical photobiont genotypes were found over a wide geographic range in three cases, A1, A2, and A3 (filled squares).

We collected 117 fresh *Nephroma* specimens from different parts of the world. In addition we obtained 158 fungal ITS sequences and 242 cyanobacterial tRNA^Leu^ (UAA) sequences of *Nephroma* specimens from NCBI GenBank. These sequences included 43 pairs of ITS and tRNA^Leu^ (UAA) sequences known to originate from the same *Nephroma* thallus (Fedrowitz et al. 2011). The total data set represented 21 different *Nephroma* species, with sequence pairs obtained from nine different *Nephroma* species. A geographic overview on our study material is shown in Fig. 2. The NCBI GenBank accession numbers with collection data are presented in Appendix S1.

### DNA extraction, amplification, and sequencing

DNA was extracted from minute thallus fragments of the lichen specimen using the GeneJET Genomic DNA Purification Kit (Fermentas, Helsinki, Finland). Amplifications of the cyanobacterial tRNA^Leu^ (UAA) intron and the fungal Internal Transcribed Spacer (ITS) region of ribosomal DNA were performed as in Fedrowitz et al. (2011). Special care was taken to ensure that both sequences came from the same lichen thallus and whenever possible both sequences were amplified from the same DNA extraction. Amplification products were purified with the GeneJET PCR Purification Kit (Fermentas). The tRNA^Leu^ (UAA) intron was sequenced with primer pair tRNA^Leu^ inF (Paulsrud and Lindblad 1998) and tRNA^Leu^ UR (Fedrowitz et al. 2011), or with a modified version of the primer tRNA^Leu^ inF (tRNA^Leu^ UFII, 5′-GGTAGACGCTACGGACTT-3′). The ITS region was sequenced with the primer pair ITS 1F and ITS 4R or ITS 5F and ITS 4R (White et al. 1990). Sequencing was performed by Macrogen Inc. in Korea or Europe. The sequence chromatograms were checked and manually edited, and the sequences were aligned using the programs BioEdit version 7.0.9.0 (Hall 1999) and PhyDE® v0.995 (Müller et al. 2005).

### Phylogenetic analysis of fungal ITS sequences

We defined the internal transcribed spacer region (ITS1 – 5.8S – ITS2, short ITS) based on sequence homology with known fungal 3′ and 5′ termini for the rns rRNA gene, the 5.8S rRNA gene, and the rnl rRNA gene (Lohtander et al. 2002; Hausner and Wang 2005). The 3′ end of the 18S rRNA (rns gene) was hence AAGGATCATTA (Lohtander et al. 2002; Hausner and Wang 2005), while the 5′ end of the rnl gene was defined as RTTGACCTCGGATCAGGTAGG.

**Figure 3:**
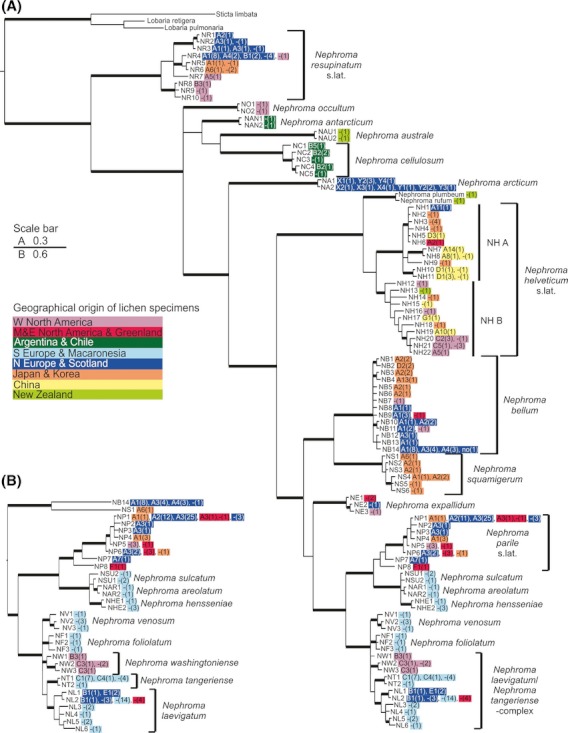
Phylogeny of the genus *Nephroma* (A) and of the *Nephroma parile* and *N. laevigatum* clades (B). The Bayesian trees were inferred from partial (A) or full (B) ITS region (493 or 513 nucleotides, respectively). Branches in bold are supported by posterior probabilities >0.94. The scale bar represents the number of substitutions per site. Genotype codes of all identified *Nostoc* genotypes are presented next to the fungal genotype codes, with “-” indicating that the associated Nostoc genotype was not available, and the number of lichen specimens representing each genotype is shown in parenthesis. Different colors denote the geographic origin of the specimen.

An alignment of 493 nucleotides including the whole 5.8S and most of the ITS1 and ITS2 regions was used in the analysis of all included *Nephroma* species, using *Lobaria pulmonaria, Lobaria retigera*, and *Sticta limbata* as outgroup (Fig. 3A). In the detailed analysis of a smaller subset of *Nephroma* species an alignment of 513 nucleotides was used which included the entire ITS region (Fig. 3B).

For Bayesian analyses the data set was divided into three sequence data partitions (partition 1: ITS1; partition 2: 5.8S and partition 3: ITS2). The best fitting nucleotide substitution models were selected by jModelTest using AIC and BIC (Posada 2008), and the GTR+I+Γ model was applied for all three regions in all analyses. The analyses were performed with MrBayes v3.1.2 (Huelsenbeck and Ronquist 2001) as described in Olsson et al. (2012). Maximum-likelihood analyses were performed with Garli v2.0 (Zwickl 2006). Six replicate maximum-likelihood analyses run with default settings resulted in trees identical in their topology (trees not shown), and recognized the same clades as those in the final trees obtained by Bayesian methods. Trees were edited using FigTree v.1.3.1 (Rambaut 2006) and CorelDRAW Graphic Suite X5.

### Haplotype analysis of cyanobacterial tRNA^Leu^ (UAA) intron sequences

Nucleotide differences in the tRNA^Leu^ (UAA) intron were used to identify different *Nostoc* genotypes using the program DnaSP v. 5.10.01 (Librado and Rozas 2009). Differences in the variable P6b stem loop of the tRNA^Leu^ (UAA) intron genotypes were checked by the prediction of a secondary folding structure using the NUPACK web server (Zadeh et al. 2011). An alignment of the entire intron sequences of all bipartite *Nephroma* species (408 nucleotide sites after alignment; 285–365 bp) was used to calculate a haplotype network using median joining in the program Network 4.6.0.0 (Bandelt et al. 1999; http://www.fluxus-engineering.com).

## Results

We obtained 112 pairs of fungal ITS and cyanobacterial tRNA^Leu^ (UAA) sequences, and five single sequences (one fungal ITS sequence and four tRNA^Leu^ (UAA) sequences) from the 117 fresh *Nephroma* specimens. After adding relevant sequences from NCBI GenBank, our data set included 155 *Nephroma* specimens from which both symbionts had been sequenced, as well as 116 single fungal ITS sequences, and 203 single cyanobacterial tRNA^Leu^ (UAA) sequences.

**Figure 4:**
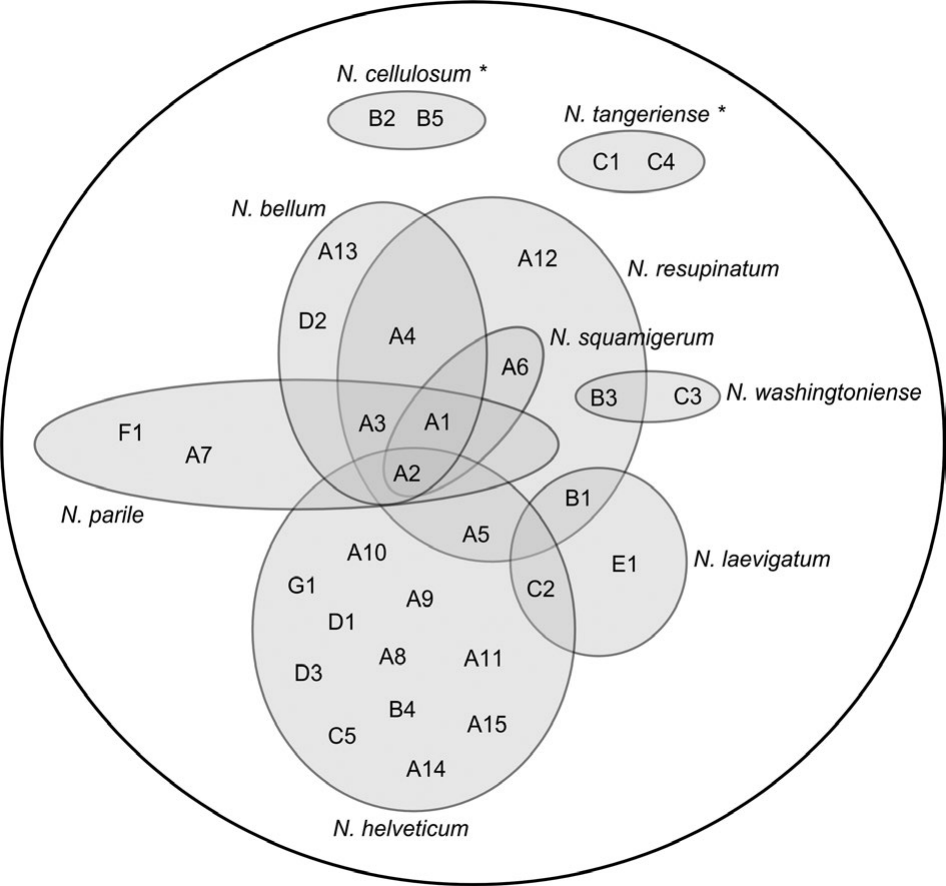
Photobiont sharing among *Nephroma* species on a global scale. Photobiont genotypes are given as letters within circles that denote fungal species with respective names given at the edge of each circle. Most bipartite *Nephroma* species share their *Nostoc* symbionts with one or more other fungal taxa, and no fungal species associates solely with a single *Nostoc* genotype, supporting the concept of functional lichen guilds. *Species for which photobiont sharing cannot be shown since only one *Nephroma* species was sampled from the same region.

Both symbiotic partners in *Nephroma* were found to show genetic variation between different geographic regions, which are described in more detail below. Several symbiont genotypes were also widely distributed, such as the fungal genotype NP1 of *Nephroma parile*, which was identified from Europe, North America, and Asia (Fig. 3), and the *Nostoc* genotype A2 which was found from North America, Europe, and Asia (Fig. 2). Most bipartite *Nephroma* species associated with *Nostoc* symbionts that were sequence identical to those of one or more other fungal taxa, and no fungal species associated solely with a single *Nostoc* genotype (Fig. 4). When comparing observed symbiont diversity patterns across spatial scales, each *Nephroma* species was found to associate with a rather restricted number of *Nostoc* genotypes locally, but with a somewhat higher number of different *Nostoc* genotypes globally (Fig. 2). However, in some cases the fungi were highly selective in their photobiont choice over vast geographical distances. For example, *N. parile* genotype NP1 was found to associate with *Nostoc* genotype A3 both in Europe and North America (Fig. 3).

### ITS-phylogeny of *Nephroma*

After removal of identical copies our alignment included 104 fungal ITS sequences from different species of *Nephroma*. The length of the ITS region in all *Nephroma* species was between 485 and 515 bp. The phylogenetic tree constructed (Fig. 3) was well in line with the recent phylogenies of *Nephroma* inferred from nuclear large subunit (nLSU), mitochondrial ribosomal small subunit (mtSSU rDNA), and ITS sequences (Lohtander et al. 2002; Sérusiaux et al. 2011). It confirmed that several species in the genus *Nephroma* can be delimited on the basis of ITS sequences alone, but also that the genus includes several heterogeneous species complexes. Several fungal genotypes were found from more than one geographic region (Fig. 3).

The well-supported *Nephroma resupinatum* s.lat. formed a sister group to all other *Nephroma* species and seems to include several presently unnamed taxa (Fig. 3A). The large *N. helveticum* s.lat. clade was divided into two internally heterogeneous but well-supported groups: *Nephroma helveticum* – complex A and *Nephroma helveticum* – complex B, respectively (Fig. 3A). The first group included specimens from northern Eurasia and eastern North America, assigned to the widely distributed (circumpolar, boreal-temperate) *Nephroma helveticum* Ach. subsp. *helveticum*. The second clade included specimens collected from temperate and tropical regions as well as NCBI GenBank sequences of lichens identified as *Nephroma tropicum* (Mull.Arg.) Zahlbr., *Nephroma isidiosum* (Nyl.) Gyelnik, and *Nephroma cellulosum* var. *isidiosum* J.S. Murray (Appendix S1). It is relevant to note that most of our Eurasian specimens of *N. helveticum* s.lat. fell into different subgroups within *N. helveticum* – complex A, and that most of our specimens from western North America belonged to one robust subgroup within *N. helveticum* – complex B. Specimens from the latter region have usually been assigned to *Nephroma helveticum* subsp. *sippeanum* (Gyelnik) Goward & Ahti.

Also *Nephroma bellum* s.lat. was divided into two robust clades: the *Nephroma bellum* – complex and *Nephroma squamigerum*, respectively (Fig. 3A). The first group included many specimens from northern Eurasia and North America, assigned to the widely distributed (circumpolar, hemiboreal to alpine) *Nephroma bellum* (Sprengel) Tuck. The second main group within *Nephroma bellum* s.lat only included specimens from East Asia: four collections from Japan and two NCBI GenBank sequences from South Korea (Fig. 3A, Appendix S1). The Japanese specimens differed from *N. bellum* in the complete lack of lichen substances that can be demonstrated by Thin-Layer-Chromatography (TLC). Also their morphology seemed to correspond to that described for *Nephroma squamigerum* Inumaru (James and White 1987). Pending the examination of type material, the East Asian taxon was tentatively named *N. squamigerum*.

In the crown group, the widely distributed *Nephroma parile* s.lat. (circumpolar, mainly northern temperate to alpine) was placed sister to a well-supported clade of three Macaronesian endemics, and the analysis indicates that further sampling within *Nephroma parile* s.lat. is likely to reveal presently unrecognized taxa (Fig. 3). Inside the *Nephroma laevigatum*/*Nephroma tangeriense* – complex, the Macaronesian endemics *N. foliolatum* and *N. venosum*, and the more widely distributed *N. tangeriense* were resolved as monophyletic, while specimens identified as ‘*Nephroma laevigatum*’ were confirmed to include specimens from two distinct taxa (Fig. 3B; Sérusiaux et al. 2011). *N. laevigatum* specimens from Europe, Macaronesia, and Eastern North America had a sister-group relationship with *N. tangeriense*, while specimens from western North America represented a distinct taxon. Without seeing type material, the latter species was tentatively named *Nephroma washingtoniense* Gyeln.

### Genetic diversity of *Nostoc* cyanobionts in *Nephroma*

**Figure 5:**
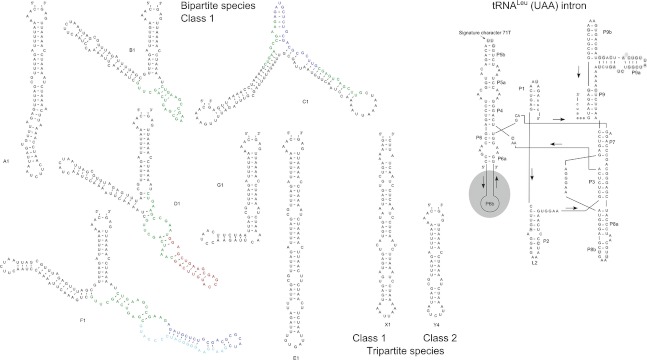
Two-dimensional model of the tRNA^Leu^ (UAA) intron secondary structure (Cech et al. 1994) and predictions of the secondary folding structures of the variable P6b stem loop for selected genotypes from bi- and tripartite species (Zadeh et al. 2011), illustrating differences in the *Nostoc* genotypes. Colored base pairs in the genotypes B1, C1, D1, and F1 show the position of major indels. Gray colored parts in the intron show variable nucleotide positions.

Our data matrix of cyanobacterial tRNA^Leu^ (UAA) intron sequences included a total of 358 sequences. These represented 40 different *Nostoc* tRNA^Leu^ (UAA) intron genotypes, of which 22 have not been reported previously (Fig. 5; Table 1).

**Table 1:** Variation in the P6b region of tRNA^Leu^ (UAA) intron sequences of *Nostoc* in *Nephroma*. The dotted line separates bipartite *Nephroma* species (genotypes A–G) from tripartite *N. arcticum* (genotypes Y and X), while the dashed line divides Class 1 from Class 2 repeat motifs. The four indel sequence elements in Class 1 are colored with their insertion points indicated by *, **, ***, or ∧∧

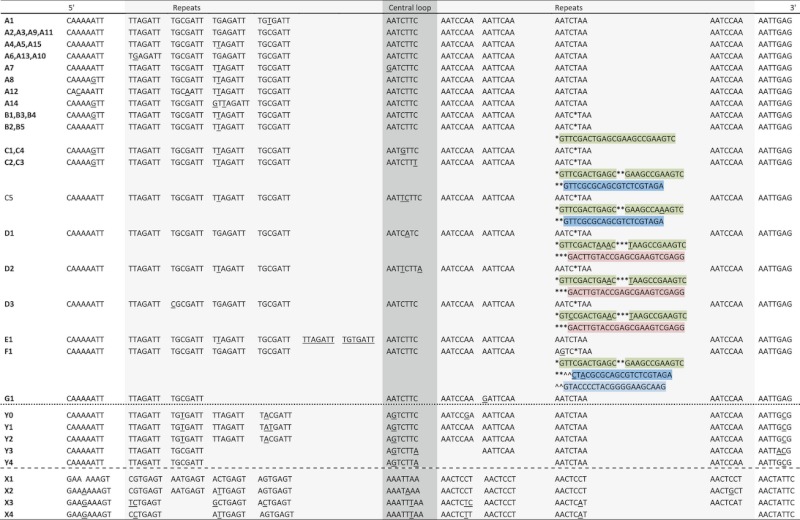

**Figure 6:**
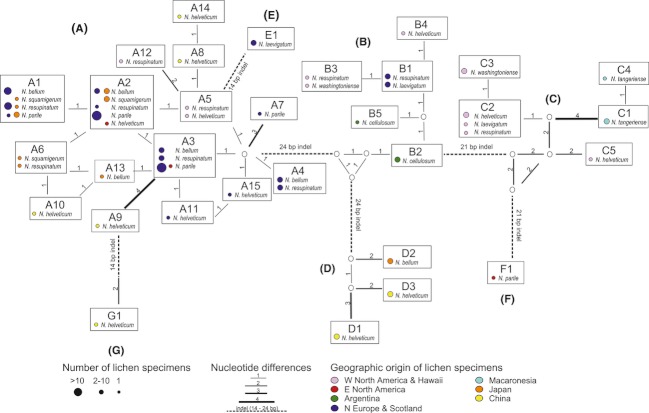
Haplotype network of *Nostoc* photobionts of bipartite *Nephroma* species from different geographic regions. The network was inferred from complete tRNA^Leu^ (UAA) sequences (285–365 nucleotides). The *Nostoc* genotypes are organized into subgroups (A–G), corresponding to the presence of major indels. Numbers by the connecting lines quantify the differences between the most similar genotypes. Major indels are shown as dashed lines.

In the haplotype network (Fig. 6) the *Nostoc* tRNA^Leu^ (UAA) intron genotypes from *Nephroma* specimens were organized into seven subgroups (A–G), corresponding to the presence of major differences in the P6b region of the tRNA^Leu^ (UAA) intron (Fig. 5; Table 1). The length of the tRNA^Leu^ (UAA) intron sequences of *Nostoc* photobionts was between 270 and 365 bp. The length variation was primarily caused by differences in the P6b region. In the transcribed intron the degenerate heptanucleotide repeats of this region base pair and fold into a hairpin structure (Fig. 5). In lichen-symbiotic *Nostoc* genotypes major differences between P6b stem loops are typically caused by different numbers of copies of repeats and, in some cases, by indels of additional sequence elements not following the heptanucleotide repeat motif. All *Nostoc* tRNA^Leu^ (UAA) intron genotypes from bipartite *Nephroma* species had a Class 1 repeat motif (Costa et al. 2002) in the P6b region, the signature character 3T in the P6b central loop, and the signature character 71T in the P5b stem loop (Table 2), confirming that they belong to a specific lineage of *Nostoc* cyanobionts which characterizes lichen species of the *Nephroma* guild (Rikkinen et al. 2002; Lohtander et al. 2003; Rikkinen 2003, 2004; Fedrowitz et al. 2011; Olsson et al. 2012). Most *Nostoc* tRNA^Leu^ (UAA) intron genotypes identified from the cephalodia of the tripartite lichen *Nephroma arcticum* also had a Class 1 repeat motif in the P6b region. However, they could be reliably differentiated from the previous group on the basis of having AGTCTTM in the P6b central loop (Table 1) and lacking the signature character 71T in the P5b region (Rikkinen 2004) (Table 2; Fig. 5). Also several other sequence characteristics helped to distinguish these *Nostoc* tRNA^Leu^ (UAA) intron genotypes from the genotypes found in all bipartite *Nephroma* species. Concurrently, the *Nostoc* genotypes of *N. arcticum* were not included in the haplotype network.

**Table 2:** Differences between bipartite and tripartite species in the P5 region of their associated *Nostoc* photobiont (underlined letters). The signal character 71T (in gray) is lacking in all tripartite species

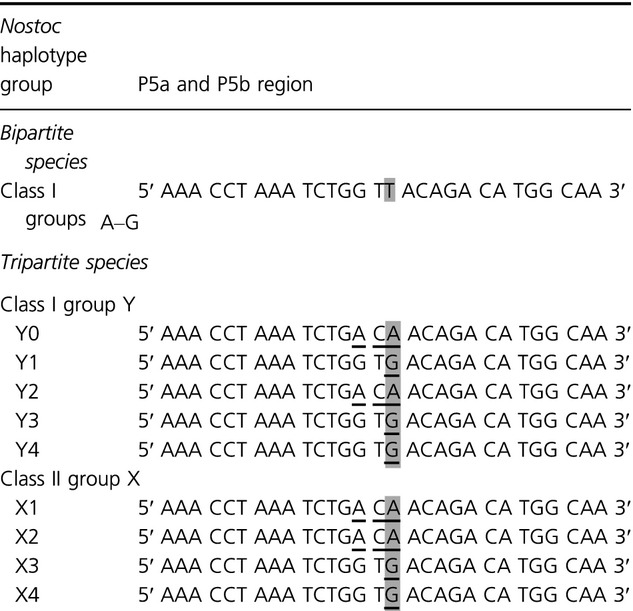

Several *Nephroma* species were found to house different *Nostoc* genotypes in different thalli within the same or different geographic regions, e.g. *Nephroma bellum* associated with cyanobacterial genotypes A1–A4 in Europe, and with A2, A13, and D2 in Japan (Figs. 6). Furthermore, in many cases several different *Nephroma* species were found to associate with the same *Nostoc* genotypes (Figs. 6). A search in NCBI GenBank and previous literature revealed that the host spectra of some *Nostoc* genotypes that were now found in bipartite *Nephroma* species (Nephromataceae) also include other species of lichen-forming fungi of the Collemataceae, Lobariaceae, and Pannariaceae (e.g., Rikkinen et al. 2002; Fedrowitz et al. 2011; Olsson et al. 2012).

The different groups of *Nostoc* genotypes in the haplotype network differed in their geographic occurrence and association with the different lichen-forming fungi (Figs. 6). Many *Nostoc* genotypes of subgroup A were common in several different *Nephroma* species, being particularly well represented in *Nephroma bellum*, *N. parile*, and *N. resupinatum*. Three *Nostoc* genotypes (A1–3) were found both from northern Europe and North America and/or East Asia, with genotype A1 being present in all above mentioned lichen lineages in both northern Europe and Japan (Figs. 6). *Nostoc* genotypes of subgroup A were also found in several specimens of *Nephroma helveticum* s.lat., originating from Asia, North America, or northern Europe (Figs. 6).

*Nostoc* genotypes of subgroup B were found in *Nephroma* thalli collected from northern Europe (including Scotland), western North America, South America, and Hawaii. They were also present in *Nephroma cellulosum* (Argentina), *N. washingtoniense* (western North America), *N. laevigatum* (northern Europe and Scotland), *N. resupinatum* s.lat. (northern Europe and western North America), and *N. helveticum* s.lat. (Hawaii). *Nostoc* genotypes of subgroup C were mainly found in thalli of *Nephroma washingtoniense* and of *N. helveticum* from western North America, but interestingly also in all specimens of *N. tangeriense* from Macaronesia (Gran Canaria). *Nostoc* genotypes of subgroup D were only found from Asia, mainly in thalli of the *Nephroma helveticum* – complex A, but also in two specimens of *N. bellum* s.lat. (Figs. 6).

While the *Nostoc* genotypes of *N. arcticum* were not included in the haplotype network they could nevertheless be organized into subgroups corresponding to the presence of major indels in the P6b region of the intron (Table 1; Fig. 5): the genotypes with Class 1 repeat motifs in the P6b region into five subgroups (Y0–Y4) and the genotypes with a Class 2 repeat motif (Costa et al. 2002) into four additional subgroups (X1–X4).

## Discussion

As pointed out by Crespo and Lumbsch (2010), the traditional approach to species recognition in lichen-forming fungi, which is largely based on thallus morphology and secondary chemistry, may have vastly underestimated the number of phylogenetic species. Many recent DNA studies have indicated that numerous distinct lineages hide under similar thallus morphologies and single species names. Concurrently, many lichen-forming fungi that have historically been thought to have nearly cosmopolitan distributions may actually represent complex aggregates of morphologically similar, but genetically distinct lineages. Our current results confirm that this is also the case in the cyanolichen genus *Nephroma*.

In fungi, the non-coding ITS region is presently regarded as the prime barcoding region (e.g., Schoch et al. 2012). While a single marker may lack sufficient resolution at the species level in some groups of lichen-forming fungi (Grube and Kroken 2000), our results confirm the early finding of Lohtander et al. (2002) that all European *Nephroma* species, as traditionally circumscribed, can be reliably identified on the basis of ITS sequences alone. Our ITS phylogeny also demonstrates the existence of several well-supported lineages within many widely distributed taxa, such as *N. parile*, *N. helveticum*, and *N. resupinatum*. While thallus morphology and secondary chemistry can undoubtedly produce additional support for the distinction of many such lineages at species level, some unnamed taxa may also have accumulated genetic divergence without unambiguous morphological disparities and thus cannot necessarily be identified using the traditional morphological concepts. Because of a limited range of morphological attributes coupled with a long evolutionary history, lichens and bryophytes might be relatively prone to evolve morphologically ‘indistinguishable’ entities that are nevertheless genetically distinct (Heinrichs et al. 2011).

Our ITS phylogeny supports the division of Asian specimens of *Nephroma bellum* s.lat. into *N. bellum* and *N. squamigerum*, respectively. It also confirms that *Nephroma laevigatum* s.lat., as traditionally circumscribed (e.g., James and White 1987; Burgaz and Martínez 1999; Vitikainen 2007; Smith et al. 2009; Esslinger 2011), represents a complex of at least two robust lineages, and supports the recognition of *N. washingtoniense* as a distinct western North American species within this complex (Sérusiaux et al. 2011). Other concurrent findings from this and previous studies (Lohtander et al. 2002; Piercey-Normore et al. 2006; Sérusiaux et al. 2011) indicate that also some other traditional taxa in the genus *Nephroma* will eventually be split into several species.

On a general level our results emphasize the need to have molecular information on both symbiotic partners before drawing far reaching conclusions about symbiont selectivity in lichens, as many presently described lichen species may actually represent heterogeneous species complexes. However, some species of *Nephroma* do appear to associate with particular *Nostoc* genotypes over a global scale (Europe–Asia, or Europe–North America). The maintenance of such specific symbiotic associations over vast geographic distances and the inherently associated long-time scales indicate that these fungi are highly selective with respect to photobiont choice. Previous studies on photobiont selectivity based on the tRNA^Leu^ (UAA) intron had found one sequence identical *Nostoc* genotype associated with *Peltigera* species in northern Europe and North America, and sequence identical *Nostoc* genotypes associated with *Pseudocyphellaria* species in New Zealand, Australia, and Chile (Paulsrud et al. 2000; Summerfield and Eaton-Rye 2006). However, sample sizes in these studies were small, and only the latter study analyzed DNA sequences of both symbionts.

The occasional breakdown of symbiotic propagules and subsequent re-association of one or both symbionts may give rise to diverse geographical mosaics of symbiont associations (Thompson 2005). In our study, several lichen taxa associated with different photobionts in different parts of its range, leading to relatively higher selectivity locally compared with lower selectivity globally. Similar patterns have previously been detected in the green algal lichen *Cladonia subtenius* (Yahr et al. 2006). It seems obvious that lichen species dispersing solely by fungal spores, such as *N. bellum*, benefit from being relatively promiscuous in their photobiont choice. In any given locality they may associate with the compatible *Nostoc* genotypes that happen to be in abundant supply. Our results show that over continental and global scales this phenomenon can sometimes even give rise to contrasting associations. For example, *N. parile* s. lat. associated exclusively with *Nostoc* genotype A1 in Japan while it mainly associated with *Nostoc* genotypes A2 or A3 in Europe and in North America. In a similar manner, *N. bellum* was commonly associated with *Nostoc* genotype A1 in Europe but associated with *Nostoc* genotype A2 in Japan. These mosaics suggest that once a certain combination of symbionts has been successfully established in a certain area, this particular combination has a tendency to become regionally dominant – possibly as a result of a similar founder effect that was previously described regarding single tree trunks in a forest landscape (Fedrowitz et al. 2011).

The presence of very closely related *Nostoc* symbionts in both *N. bellum* and *N. squamigerum* could indicate that an association to specific lineages of symbiotic cyanobacteria can be maintained across speciation events in lichen-forming fungi. However, examples of widespread cyanobiont sharing are much more common. For example, the fungi in several clades of *N. resupinatum* s. lat. were found to share identical *Nostoc* symbionts with fungal species in several distantly related taxa (*N. bellum*, *N. helveticum*, *N. laevigatum*, *N. parile)*. In addition to the different *Nephroma* species, the fungal hosts of some *Nostoc* symbionts are known to also include species from other genera of lichen-forming fungi (Rikkinen et al. 2002; Fedrowitz et al. 2011; Olsson et al. 2012).

Our results indicate that the present distribution of some indel types in the P6b stem loops of *Nostoc* symbionts may reflect dispersal history. For example, the bimodal distribution of one particular indel type (‘group C’, Figs. 6) indicates a close relationship between the *Nostoc* symbionts of the Macaronesian and western Mediterranean *N. tangeriense* and western North American lichens, including *N. washingtoniense*. Such a connection was proposed by Sérusiaux et al. (2011) on the basis of fungal phylogeny and interpreted as evidence of long-distance dispersal from Macaronesia to western North America. If this hypothesis is correct, our findings propose a joint dispersal of the *Nostoc* symbiont with its fungal host, presumably within a specialized symbiotic diaspore, or within a thallus fragment. In North America, the *Nostoc* cyanobiont could then have been subsequently recruited into several other lichen symbioses as suggested by the present diversity of different fungal hosts.

The two tripartite *Nephroma* species in northern Europe (*N. arcticum* and *N. expallidum*) do not form a monophyletic lineage, but both house *Nostoc* symbionts clearly distinct from those typically found in bipartite *Nephroma* species (Lohtander et al. 2002). Our study confirms this and adds that not only Class 1 but also Class 2 repeat motifs are found in *N. arcticum* (Paulsrud and Lindblad 1998; Paulsrud et al. 1998). The clear distinction of photobiont types between bi- and tripartite species supports the view that the evolution of tripartite *Nephroma* species cannot have occurred simply via an additional association with a green algal photobiont (*Coccomyxa*). Instead, any evolutionary transition between the two symbiosis types must have required a concurrent switch of *Nostoc* symbionts (Lohtander et al. 2003).

In conclusion, our results illustrate that all bipartite *Nephroma* species associate with one group of *Nostoc* different from the group of *Nostoc* typically found in tripartite *Nephroma* species. According to all available evidence, this association must have been inherited from the common ancestor of all extant bipartite species, and the association as such is highly conserved. As the tripartite *Nephroma* species are polyphyletic and always associate with a different group of *Nostoc*, evolutionary transitions between the two types of symbioses must have occurred repeatedly in *Nephroma* and these processes have invariably involved a switch from one lineage of *Nostoc* symbionts to another. Both symbiotic partners in *Nephroma* show genetic differentiation between geographical regions, but several symbiont genotypes or genotype groups are widely distributed, and their present distributions may reflect species dispersal history. Even particular associations between specific symbiotic genotypes are sometimes maintained over vast geographical distances. Most if not all bipartite *Nephroma* species share their *Nostoc* symbionts with one or more other fungus, and no species associates solely with a single *Nostoc* genotype. This supports the concept of functional guilds in which fungal species rely on a common pool of closely related *Nostoc* genotypes. As a whole, the symbiont selectivity patterns within *Nephroma* are best described as geographic mosaics, with higher selectivity locally than globally. This might often reflect specific habitat preferences of particular symbiont combinations, but also the influence of founder effects.

## Supporting Information

**Appendix S1.** Voucher information, associated NCBI GenBank accession numbers, and genotype names for the fungal ITS and the cyanobacterial tRNA^Leu^ (UAA) sequences used in the study.
