# Supplementary material for: Geographic mosaic of symbiont selectivity in a genus of epiphytic cyanolichens
Source: Ecol Evol. 2012 Aug 10;2(9):2291–303. doi: 10.1002/ece3.343 (PMC3488679; doi:10.1002/ece3.343)
Supplement: Supplementary file 1 [file ece30002-2291-SD1.doc]

**SUPPORTING INFORMATION**

Geographic mosaic of symbiont selectivity in a genus of epiphytic cyanolichens.

Katja Fedrowitz, Ulla Kaasalainen, Jouko Rikkinen

**Appendix S1** Voucher information, associated GenBank accession numbers, and genotype names for the fungal ITS and the cyanobacterial tRNA^Leu^ (UAA) sequences used in the study. New accessions are highlighted in grey.

|  | |  | | **Genotypes** | | | **GenBank accession numbers** | | | |
| --- | --- | --- | --- | --- | --- | --- | --- | --- | --- | --- |
|  | **Species** | | **Voucher** | | **ITS** | **tRNA^Leu^ (UAA)** | | **ITS** | **tRNA^Leu^ (UAA)** |  |
|  | *Lobaria pulmonaria* | | Finland (Fedrowitz et al. 2011) | | Lobaria pulmonaria | ----- | | HM448799 | ----- |  |
|  | *Lobaria retigera* | | China (J. Rikkinen U549) | | Lobaria retigera | ----- | | JN857197 | JN857078 |  |
|  | *Nephroma antarcticum* | | Chile (Piercey-Normore et al. 2006) | | NAN1 | ----- | | DQ320563 | ----- |  |
|  | *N. antarcticum* | | Argentina (Stenroos et al. 2003) | | NAN2 | ----- | | AF524897 | ----- |  |
|  | *Nephroma arcticum* | | Sweden (Paulsrud & Lindblad 1998) | | ----- | Y0 | | ----- | AF019918 |  |
|  | *N. arcticum* | | Sweden (Paulsrud & Lindblad 1998) | | ----- | Y2 | | ----- | AF019912 |  |
|  | *N. arcticum* | | Sweden (Paulsrud & Lindblad 1998) | | ----- | Y3 | | ----- | AF019916 |  |
|  | *N. arcticum* | | Sweden (Paulsrud & Lindblad 1998) | | ----- | Y3 | | ----- | AF019917 |  |
|  | *N. arcticum* | | Finland (Paulsrud et al. 1998) | | ----- | Y3 | | ----- | AF055659 |  |
|  | *N. arcticum* | | Finland (J. Rikkinen JR09_221_1) | | NA1 | Y2 | | JN857198 | JN857079 |  |
|  | *N. arcticum* | | Finland (J. Rikkinen JR09_228B_1) | | NA1 | Y2 | | JN857199 | JN857080 |  |
|  | *N. arcticum* | | Finland (J. Rikkinen JR09_230B_1) | | NA1 | Y2 | | JN857200 | JN857081 |  |
|  | *N. arcticum* | | Sweden (K. Fedrowitz U432) | | NA1 | Y4 | | JN857201 | JN857082 |  |
|  | *N. arcticum* | | Finland (J. Rikkinen U564b) | | NA1 | X1 | | JN857202 | JN857083 |  |
|  | *N. arcticum* | | Finland (J. Rikkinen JR09_210_1) | | NA2 | Y1 | | JN857203 | JN857084 |  |
|  | *N. arcticum* | | Finland (J. Rikkinen JR09_228A_1) | | NA2 | Y2 | | JN857204 | JN857085 |  |
|  | *N. arcticum* | | Finland (J. Rikkinen K_J52) | | NA2 | Y2 | | JN857205 | JN857086 |  |
|  | *N. arcticum* | | Finland (J. Rikkinen JR09_230A_1) | | NA2 | Y3 | | JN857206 | JN857087 |  |
|  | *N. arcticum* | | Finland (J. Rikkinen K_J58) | | NA2 | X2 | | JN857207 | JN857088 |  |
|  | *N. arcticum* | | Norway (U. Kaasalainen U369) | | NA2 | X3 | | JN857208 | JN857089 |  |
|  | *N. arcticum* | | Finland (J. Rikkinen K_J45) | | NA2 | X4 | | JN857209 | JN857090 |  |
|  | *Nephroma areolatum* | | Madeira (Serusiaux et al. 2011) | | NAR1 | ----- | | HQ455057 | ----- |  |
|  | *N. areolatum* | | Madeira (Serusiaux et al. 2011) | | NAR2 | ----- | | HQ455056 | ----- |  |
|  | *Nephroma australe* | | New Zealand (Lohtander et al. 2002) | | NAU1 | ----- | | AY124115 | ----- |  |
|  | *N. australe* | | New Zealand(?) (Unpublished) | | NAU2 | ----- | | AF347699 | ----- |  |
|  | *Nephroma bellum* | | Finland (Fedrowitz et al. 2011) | | ----- | A1 | | ----- | HM448483 |  |
|  | *N. bellum* | | Finland (Fedrowitz et al. 2011) | | ----- | A1 | | ----- | HM448488 |  |
|  | *N. bellum* | | Finland (Fedrowitz et al. 2011) | | ----- | A1 | | ----- | HM448490 |  |
|  | *N. bellum* | | Finland (Fedrowitz et al. 2011) | | ----- | A1 | | ----- | HM448491 |  |
|  | *N. bellum* | | Finland (Fedrowitz et al. 2011) | | ----- | A1 | | ----- | HM448494 |  |
|  | *N. bellum* | | Finland (Fedrowitz et al. 2011) | | ----- | A1 | | ----- | HM448496 |  |
|  | *N. bellum* | | Finland (Fedrowitz et al. 2011) | | ----- | A1 | | ----- | HM448497 |  |
|  | *N. bellum* | | Finland (Fedrowitz et al. 2011) | | ----- | A1 | | ----- | HM448498 |  |
|  | *N. bellum* | | Finland (Fedrowitz et al. 2011) | | ----- | A1 | | ----- | HM448500 |  |
|  | *N. bellum* | | Finland (Fedrowitz et al. 2011) | | ----- | A1 | | ----- | HM448501 |  |
|  | *N. bellum* | | Finland (Fedrowitz et al. 2011) | | ----- | A1 | | ----- | HM448502 |  |
|  | *N. bellum* | | Finland (Fedrowitz et al. 2011) | | ----- | A1 | | ----- | HM448503 |  |
|  | *N. bellum* | | Finland (Fedrowitz et al. 2011) | | ----- | A1 | | ----- | HM448504 |  |
|  | *N. bellum* | | Finland (Fedrowitz et al. 2011) | | ----- | A1 | | ----- | HM448505 |  |
|  | *N. bellum* | | Finland (Fedrowitz et al. 2011) | | ----- | A1 | | ----- | HM448506 |  |
|  | *N. bellum* | | Finland (Fedrowitz et al. 2011) | | ----- | A1 | | ----- | HM448507 |  |
|  | *N. bellum* | | Finland (Fedrowitz et al. 2011) | | ----- | A1 | | ----- | HM448508 |  |
|  | *N. bellum* | | Finland (Fedrowitz et al. 2011) | | ----- | A1 | | ----- | HM448509 |  |
|  | *N. bellum* | | Finland (Fedrowitz et al. 2011) | | ----- | A1 | | ----- | HM448510 |  |
|  | *N. bellum* | | Finland (Fedrowitz et al. 2011) | | ----- | A1 | | ----- | HM448512 |  |
|  | *N. bellum* | | Finland (Fedrowitz et al. 2011) | | ----- | A1 | | ----- | HM448515 |  |
|  | *N. bellum* | | Finland (Fedrowitz et al. 2011) | | ----- | A1 | | ----- | HM448518 |  |
|  | *N. bellum* | | Finland (Fedrowitz et al. 2011) | | ----- | A1 | | ----- | HM448519 |  |
|  | *N. bellum* | | Finland (Fedrowitz et al. 2011) | | ----- | A1 | | ----- | HM448520 |  |
|  | *N. bellum* | | Finland (Fedrowitz et al. 2011) | | ----- | A1 | | ----- | HM448521 |  |
|  | *N. bellum* | | Finland (Fedrowitz et al. 2011) | | ----- | A1 | | ----- | HM448522 |  |
|  | *N. bellum* | | Finland (Fedrowitz et al. 2011) | | ----- | A1 | | ----- | HM448523 |  |
|  | *N. bellum* | | Finland (Fedrowitz et al. 2011) | | ----- | A1 | | ----- | HM448524 |  |
|  | *N. bellum* | | Finland (Fedrowitz et al. 2011) | | ----- | A1 | | ----- | HM448525 |  |
|  | *N. bellum* | | Finland (Fedrowitz et al. 2011) | | ----- | A1 | | ----- | HM448526 |  |
|  | *N. bellum* | | Finland (Fedrowitz et al. 2011) | | ----- | A1 | | ----- | HM448527 |  |
|  | *N. bellum* | | Finland (Fedrowitz et al. 2011) | | ----- | A1 | | ----- | HM448528 |  |
|  | *N. bellum* | | Finland (Fedrowitz et al. 2011) | | ----- | A1 | | ----- | HM448529 |  |
|  | *N. bellum* | | Finland (Fedrowitz et al. 2011) | | ----- | A1 | | ----- | HM448530 |  |
|  | *N. bellum* | | Finland (Fedrowitz et al. 2011) | | ----- | A1 | | ----- | HM448531 |  |
|  | *N. bellum* | | Finland (Fedrowitz et al. 2011) | | ----- | A1 | | ----- | HM448532 |  |
|  | *N. bellum* | | Finland (Fedrowitz et al. 2011) | | ----- | A1 | | ----- | HM448533 |  |
|  | *N. bellum* | | Finland (Fedrowitz et al. 2011) | | ----- | A1 | | ----- | HM448534 |  |
|  | *N. bellum* | | Finland (Fedrowitz et al. 2011) | | ----- | A1 | | ----- | HM448536 |  |
|  | *N. bellum* | | Finland (Fedrowitz et al. 2011) | | ----- | A1 | | ----- | HM448537 |  |
|  | *N. bellum* | | Finland (Fedrowitz et al. 2011) | | ----- | A1 | | ----- | HM448538 |  |
|  | *N. bellum* | | Finland (Fedrowitz et al. 2011) | | ----- | A1 | | ----- | HM448539 |  |
|  | *N. bellum* | | Finland (Fedrowitz et al. 2011) | | ----- | A1 | | ----- | HM448540 |  |
|  | *N. bellum* | | Finland (Fedrowitz et al. 2011) | | ----- | A1 | | ----- | HM448541 |  |
|  | *N. bellum* | | Finland (Fedrowitz et al. 2011) | | ----- | A1 | | ----- | HM448542 |  |
|  | *N. bellum* | | Finland (Fedrowitz et al. 2011) | | ----- | A1 | | ----- | HM448543 |  |
|  | *N. bellum* | | Finland (Fedrowitz et al. 2011) | | ----- | A1 | | ----- | HM448544 |  |
|  | *N. bellum* | | Finland (Fedrowitz et al. 2011) | | ----- | A1 | | ----- | HM448545 |  |
|  | *N. bellum* | | Finland (Fedrowitz et al. 2011) | | ----- | A1 | | ----- | HM448546 |  |
|  | *N. bellum* | | Finland (Fedrowitz et al. 2011) | | ----- | A1 | | ----- | HM448547 |  |
|  | *N. bellum* | | Finland (Fedrowitz et al. 2011) | | ----- | A1 | | ----- | HM448548 |  |
|  | *N. bellum* | | Finland (Fedrowitz et al. 2011) | | ----- | A1 | | ----- | HM448549 |  |
|  | *N. bellum* | | Finland (Fedrowitz et al. 2011) | | ----- | A1 | | ----- | HM448550 |  |
|  | *N. bellum* | | Finland (Fedrowitz et al. 2011) | | ----- | A1 | | ----- | HM448551 |  |
|  | *N. bellum* | | Finland (Fedrowitz et al. 2011) | | ----- | A1 | | ----- | HM448552 |  |
|  | *N. bellum* | | Finland (Fedrowitz et al. 2011) | | ----- | A1 | | ----- | HM448553 |  |
|  | *N. bellum* | | Finland (Fedrowitz et al. 2011) | | ----- | A1 | | ----- | HM448554 |  |
|  | *N. bellum* | | Finland (Fedrowitz et al. 2011) | | ----- | A1 | | ----- | HM448555 |  |
|  | *N. bellum* | | Finland (Fedrowitz et al. 2011) | | ----- | A1 | | ----- | HM448556 |  |
|  | *N. bellum* | | Finland (Fedrowitz et al. 2011) | | ----- | A1 | | ----- | HM448557 |  |
|  | *N. bellum* | | Finland (Fedrowitz et al. 2011) | | ----- | A1 | | ----- | HM448558 |  |
|  | *N. bellum* | | Finland (Fedrowitz et al. 2011) | | ----- | A1 | | ----- | HM448559 |  |
|  | *N. bellum* | | Finland (Fedrowitz et al. 2011) | | ----- | A1 | | ----- | HM448560 |  |
|  | *N. bellum* | | Finland (Fedrowitz et al. 2011) | | ----- | A1 | | ----- | HM448561 |  |
|  | *N. bellum* | | Finland (Fedrowitz et al. 2011) | | ----- | A1 | | ----- | HM448562 |  |
|  | *N. bellum* | | Finland (Fedrowitz et al. 2011) | | ----- | A1 | | ----- | HM448563 |  |
|  | *N. bellum* | | Finland (Fedrowitz et al. 2011) | | ----- | A4 | | ----- | HM448698 |  |
|  | *N. bellum* | | Finland (Fedrowitz et al. 2011) | | ----- | A4 | | ----- | HM448700 |  |
|  | *N. bellum* | | Finland (Rikkinen et al. 2002) | | ----- | A1 | | ----- | AF509360 |  |
|  | *N. bellum* | | Finland (Rikkinen et al. 2002) | | ----- | A1 | | ----- | AF509361 |  |
|  | *N. bellum* | | Finland (Rikkinen et al. 2002) | | ----- | A1 | | ----- | AF509362 |  |
|  | *N. bellum* | | Finland (Rikkinen et al. 2002) | | ----- | A1 | | ----- | AF509363 |  |
|  | *N. bellum* | | Finland (Rikkinen et al. 2002) | | ----- | A1 | | ----- | AF509364 |  |
|  | *N. bellum* | | Finland (Rikkinen et al. 2002) | | ----- | A1 | | ----- | AF509365 |  |
|  | *N. bellum* | | Finland (Rikkinen et al. 2002) | | ----- | A1 | | ----- | AF509366 |  |
|  | *N. bellum* | | Finland (Rikkinen et al. 2002) | | ----- | A1 | | ----- | AF509367 |  |
|  | *N. bellum* | | Finland (Rikkinen et al. 2002) | | ----- | A1 | | ----- | AF509368 |  |
|  | *N. bellum* | | Finland (Rikkinen et al. 2002) | | ----- | A1 | | ----- | AF509369 |  |
|  | *N. bellum* | | Finland (Rikkinen et al. 2002) | | ----- | A1 | | ----- | AF509370 |  |
|  | *N. bellum* | | Japan (G. Thor (GT 24040) (KU494b)) | | NB1 | A2 | | JN857210 | JN857091 |  |
|  | *N. bellum* | | Japan (G. Thor (GT 24041) (KU519)) | | NB1 | A2 | | JN857211 | JN857092 |  |
|  | *N. bellum* | | Japan (G. Thor (400A4/2nb) (KU518)) | | NB2 | D2 | | JN857212 | JN857093 |  |
|  | *N. bellum* | | Japan (G. Thor (GT 24333) (KU559)) | | NB2 | D2 | | JN857213 | JN857094 |  |
|  | *N. bellum* | | Japan (G. Thor (600A4/1) (KU557a)) | | NB3 | A2 | | JN857214 | JN857095 |  |
|  | *N. bellum* | | Japan (G. Thor (GT 24213) (KU558)) | | NB3 | A2 | | JN857215 | JN857096 |  |
|  | *N. bellum* | | Japan (A. Frisch (10/Jp3) (KU523a)) | | NB4 | A13 | | JN857216 | JN857097 |  |
|  | *N. bellum* | | Japan (A. Frisch (10/Jp5) (KU525a)) | | NB5 | A2 | | JN857217 | JN857098 |  |
|  | *N. bellum* | | Japan (G. Thor (GT 24356) (KU560)) | | NB6 | A2 | | JN857218 | JN857099 |  |
|  | *N. bellum* | | Canada SW (Lohtander et al. 2002) | | NB7 | ----- | | AY124131 | ----- |  |
|  | *N. bellum* | | Sweden (A. Frisch (AF1nb) (KU491)) | | NB8 | A1 | | JN857219 | JN857100 |  |
|  | *N. bellum* | | Canada, BC (Serusiaux et al. 2011) | | NB9 | ----- | | HQ455058 | ----- |  |
|  | *N. bellum* | | Finland (J. Rikkinen (JR08S1T) (KUJ119)) | | NB9 | A1 | | JN857220 | JN857101 |  |
|  | *N. bellum* | | Finland (J. Rikkinen (JR08S1U) (KUJ120)) | | NB9 | A1 | | JN857221 | JN857102 |  |
|  | *N. bellum* | | Finland (J. Rikkinen (JR201006A-C) (KU505)) | | NB9 | A1 | | JN857222 | JN857103 |  |
|  | *N. bellum* | | Finland (Fedrowitz et al. 2011) | | NB10 | A1 | | HM448749 | HM448495 |  |
|  | *N. bellum* | | Finland (Fedrowitz et al. 2011) | | NB10 | A2 | | HM448754 | HM448614 |  |
|  | *N. bellum* | | Finland (Fedrowitz et al. 2011) | | NB10 | A2 | | HM448753 | HM448615 |  |
|  | *N. bellum* | | Finland (Fedrowitz et al. 2011) | | NB11 | A1 | | HM448746 | HM448485 |  |
|  | *N. bellum* | | Finland (Fedrowitz et al. 2011) | | NB11 | A1 | | HM448744 | HM448535 |  |
|  | *N. bellum* | | USA, Oregon (Lohtander et al. 2002) | | NB11 | ----- | | AY124130 | ----- |  |
|  | *N. bellum* | | Sweden (K. Fedrowitz KU490) | | NB12 | A3 | | JN857223 | JN857104 |  |
|  | *N. bellum* | | Finland (Fedrowitz et al. 2011) | | NB13 | A1 | | HM448748 | HM448516 |  |
|  | *N. bellum* | | Finland (Fedrowitz et al. 2011) | | NB14 | A1 | | HM448745 | HM448482 |  |
|  | *N. bellum* | | Finland (Fedrowitz et al. 2011) | | NB14 | A1 | | HM448747 | HM448484 |  |
|  | *N. bellum* | | Finland (Fedrowitz et al. 2011) | | NB14 | A1 | | HM448750 | HM448486 |  |
|  | *N. bellum* | | Finland (Fedrowitz et al. 2011) | | NB14 | A1 | | HM448751 | HM448487 |  |
|  | *N. bellum* | | Finland (Fedrowitz et al. 2011) | | NB14 | A1 | | HM448752 | HM448489 |  |
|  | *N. bellum* | | Finland (J. Rikkinen (JR201004) (KU503)) | | NB14 | A1 | | JN857224 | JN857105 |  |
|  | *N. bellum* | | Finland (J. Rikkinen (JR201009A-C) (KU506)) | | NB14 | A1 | | JN857225 | JN857106 |  |
|  | *N. bellum* | | Finland (J. Rikkinen (JR201009A-C) (KU507)) | | NB14 | A1 | | JN857226 | JN857107 |  |
|  | *N. bellum* | | Finland (J. Rikkinen (JR08S1S) (KUJ118)) | | NB14 | A3 | | JN857227 | JN857108 |  |
|  | *N. bellum* | | Finland (J. Rikkinen (JR201005) (KU504)) | | NB14 | A3 | | JN857228 | JN857109 |  |
|  | *N. bellum* | | Finland (J. Rikkinen (JR201007A-C) (KU514)) | | NB14 | A3 | | JN857229 | JN857110 |  |
|  | *N. bellum* | | Sweden (K. Fedrowitz KU489) | | NB14 | A3 | | JN857230 | JN857111 |  |
|  | *N. bellum* | | Finland (Fedrowitz et al. 2011) | | NB14 | A4 | | HM448755 | HM448697 |  |
|  | *N. bellum* | | Finland (Fedrowitz et al. 2011) | | NB14 | A4 | | HM448756 | HM448699 |  |
|  | *N. bellum* | | Finland (Fedrowitz et al. 2011) | | NB14 | A4 | | HM448757 | HM448701 |  |
|  | *N. bellum* | | Finland (Lohtander et al. 2002) | | NB14 | ----- | | AY124133 | ----- |  |
|  | *Nephroma cellulosum* | | Argentina (K. Fedrowitz AR39) | | NC1 | B5 | | JN857231 | JN857115 |  |
|  | *N. cellulosum* | | Argentina (K. Fedrowitz AR63) | | NC2 | B2 | | JN857232 | JN857112 |  |
|  | *N. cellulosum* | | Argentina (K. Fedrowitz AR71) | | NC2 | B2 | | JN857233 | JN857113 |  |
|  | *N. cellulosum* | | Argentina (Lohtander et al. 2002) | | NC3 | ----- | | AY124117 | ----- |  |
|  | *N. cellulosum* | | Argentina (K. Fedrowitz AR87) | | NC4 | B2 | | JN857234 | JN857114 |  |
|  | *N. cellulosum* | | Argentina (Lohtander et al. 2002) | | NC5 | ----- | | AY124116 | ----- |  |
|  | *Nephroma expallidum* | | Canada, Manitoba (Piercey-Normore et al. 2006) | | NE1 | ----- | | DQ320561 | ----- |  |
|  | *N. expallidum* | | Greenland (Lohtander et al. 2002) | | NE1 | ----- | | AY124134 | ----- |  |
|  | *N. expallidum* | | Norway (Lohtander et al. 2002) | | NE2 | ----- | | AY124135 | ----- |  |
|  | *N. expallidum* | | Canada SW (Lohtander et al. 2002) | | NE3 | ----- | | AY124136 | ----- |  |
|  | *Nephroma foliolatum* | | Madeira (Serusiaux et al. 2011) | | NF1 | ----- | | HQ455066 | ----- |  |
|  | *N. foliolatum* | | Madeira (Serusiaux et al. 2011) | | NF2 | ----- | | HQ455065 | ----- |  |
|  | *N. foliolatum* | | Madeira (Serusiaux et al. 2011) | | NF3 | ----- | | HQ455061 | ----- |  |
|  | *Nephroma helveticum* | | China (J. Rikkinen (65) (KU529)) | | ----- | A14 | | ----- | JN857116 |  |
|  | *N. helveticum* | | China (Ahti (46350)_TLC 88/97 (KU1005)) | | ----- | A9 | | ----- | JN857117 |  |
|  | *N. helveticum* | | China, Hunan (Rikkinen et al. 2002) | | ----- | D3 | | ----- | AF509407 |  |
|  | *N. helveticum* | | Finland, Oulanka (P. Halonen KU534) | | ----- | A15 | | ----- | JN857118 |  |
|  | *N. helveticum* | | Hawaii (Weber & Randolph *N. tropicum* (KU1006)) | | ----- | B4 | | ----- | JN857119 |  |
|  | *N. helveticum* | | USA, Oregon (Rikkinen et al. 2002) | | ----- | C2 | | ----- | AF509402 |  |
|  | *N. helveticum* | | Russia, Paanajärvi NP (P. Halonen (KU535)) | | NH1 | A11 | | JN857235 | JN857120 |  |
|  | *N. helveticum* | | South Korea (Unpublished) | | NH2 | ----- | | DQ066707 | ----- |  |
|  | *N. helveticum* | | South Korea (Unpublished) | | NH3 | ----- | | AY826402 | ----- |  |
|  | *N. helveticum* | | South Korea (Unpublished) | | NH3 | ----- | | DQ066703 | ----- |  |
|  | *N. helveticum* | | South Korea (Unpublished) | | NH3 | ----- | | DQ066704 | ----- |  |
|  | *N. helveticum* | | South Korea (Unpublished) | | NH3 | ----- | | DQ066706 | ----- |  |
|  | *N. helveticum* | | South Korea (Unpublished) | | NH4 | ----- | | DQ066705 | ----- |  |
|  | *N. helveticum* | | NE China (ITS: Lohtander et al. 2002) (ITS & tRNALeu: KU533) | | NH5 | D3 | | AY124127 & JN857236 | JN857121 |  |
|  | *N. helveticum* | | Canada QC (H. Coffey & C. Freebury KU1002) | | NH6 | A2 | | JN857237 | JN857122 |  |
|  | *N. helveticum* | | China (J. Rikkinen (734) (KU530)) | | NH7 | A14 | | JN857238 | JN857123 |  |
|  | *N. helveticum* | | China (J. Rikkinen (JR000728, DNA5) (KU513)) | | NH8 | A8 | | JN857239 | JN857124 |  |
|  | *N. helveticum* | | China (Lohtander et al. 2002) | | NH8 | ----- | | AY124118 | ----- |  |
|  | *N. helveticum* | | South Korea (Unpublished) | | NH9 | ----- | | DQ066702 | ----- |  |
|  | *N. helveticum* | | China (J. Rikkinen (JR000326, DNA6) (KU512a)) | | NH10 | D1 | | JN857240 | JN857125 |  |
|  | *N. helveticum* | | China (Lohtander et al. 2002) | | NH10 | ----- | | AY124129 | ----- |  |
|  | *N. helveticum* | | China (J. Rikkinen (3575) (KU532)) | | NH11 | D1 | | JN857241 | JN857126 |  |
|  | *N. helveticum* | | China (J. Rikkinen (990626) (KU528)) | | NH11 | D1 | | JN857242 | JN857127 |  |
|  | *N. helveticum* | | China (J. Rikkinen (JR000119, DNA8) (KU510)) | | NH11 | D1 | | JN857243 | JN857128 |  |
|  | *N. helveticum* | | China (Lohtander et al. 2002) | | NH11 | ----- | | AY124122 | ----- |  |
|  | *N. helveticum* | | USA, SW (Lohtander et al. 2002) | | NH12 | ----- | | AY124119 | ----- |  |
|  | *N. helveticum* | | New Zealand (?) (Unpublished) *N. cellulosum var. isidioferum* | | NH13 | ----- | | AF350287 | ----- |  |
|  | *N. helveticum* | | South Korea (?) (Unpublished) *N. tropicum* | | NH14 | ----- | | DQ066712 | ----- |  |
|  | *N. helveticum* | | China (Unpublished) | | NH15 | ----- | | DQ001292 | ----- |  |
|  | *N. helveticum* | | Canada, BC (Piercey-Normore et al. 2006) *N. isidiosum* | | NH16 | ----- | | DQ320562 | ----- |  |
|  | *N. helveticum* | | NW China (ITS: Lohtander et al. 2002) (tRNALeu: KU1007) | | NH17 | G1 | | AY124121 | JN857129 |  |
|  | *N. helveticum* | | South Korea (?) (Unpublished) *N. tropicum* | | NH18 | ----- | | DQ066711 | ----- |  |
|  | *N. helveticum* | | S China (Tibet) (ITS: Lohtander et al. 2002) (tRNALeu: KU1004) | | NH19 | A10 | | AY124120 | JN857130 |  |
|  | *N. helveticum* | | USA, Oregon (J. Rikkinen (JR98A32) (KU536)) | | NH20 | C2 | | JN857244 | JN857131 |  |
|  | *N. helveticum* | | USA, Oregon (J. Rikkinen (JR98A34A) (KU538)) | | NH20 | C2 | | JN857245 | JN857132 |  |
|  | *N. helveticum* | | USA, Oregon (J. Rikkinen (JR98A34B) (KU539)) | | NH20 | C2 | | JN857246 | JN857133 |  |
|  | *N. helveticum* | | USA, Oregon (Lohtander et al. 2002) | | NH20 | ----- | | AY124124 | ----- |  |
|  | *N. helveticum* | | USA, Oregon (U. Kaasalainen U147) | | NH21 | C5 | | JN857247 | JN857134 |  |
|  | *N. helveticum* | | USA, Oregon (Lohtander et al. 2002) | | NH21 | ----- | | AY124123 | ----- |  |
|  | *N. helveticum* | | USA, Oregon (Lohtander et al. 2002) | | NH21 | ----- | | AY124125 | ----- |  |
|  | *N. helveticum* | | USA, Oregon (Lohtander et al. 2002) | | NH21 | ----- | | AY124126 | ----- |  |
|  | *N. helveticum* | | USA, California (U. Kaasalainen U180) | | NH22 | A5 | | JN857248 | JN857135 |  |
|  | *Nephroma hensseniae* | | Azores (Serusiaux et al. 2011) | | NHE1 | ----- | | HQ455073 | ----- |  |
|  | *N. hensseniae* | | Azores (Serusiaux et al. 2011) | | NHE2 | ----- | | HQ455072 | ----- |  |
|  | *N. hensseniae* | | Azores (Serusiaux et al. 2011) | | NHE2 | ----- | | HQ455074 | ----- |  |
|  | *N. hensseniae* | | Azores (Serusiaux et al. 2011) | | NHE2 | ----- | | HQ455075 | ----- |  |
|  | *Nephroma laevigatum* | | USA, Oregon (Rikkinen et al. 2002) | | ----- | C2 | | ----- | AF509403 |  |
|  | *N. laevigatum* | | Scotland (K. Fedrowitz KUS30) | | NL1 | E1 | | JN857249 | JN857136 |  |
|  | *N. laevigatum* | | Scotland (K. Fedrowitz KUS37) | | NL1 | B1 | | JN857250 | JN857137 |  |
|  | *N. laevigatum* | | Scotland (K. Fedrowitz KUS39) | | NL1 | E1 | | JN857251 | JN857138 |  |
|  | *N. laevigatum* | | Azores (Serusiaux et al. 2011) | | NL2 | ----- | | HQ455082 | ----- |  |
|  | *N. laevigatum* | | Azores (Serusiaux et al. 2011) | | NL2 | ----- | | HQ455083 | ----- |  |
|  | *N. laevigatum* | | Azores (Serusiaux et al. 2011) | | NL2 | ----- | | HQ455084 | ----- |  |
|  | *N. laevigatum* | | Azores (Serusiaux et al. 2011) | | NL2 | ----- | | HQ455085 | ----- |  |
|  | *N. laevigatum* | | Canada, Newfoundland (Serusiaux et al. 2011) | | NL2 | ----- | | HQ455095 | ----- |  |
|  | *N. laevigatum* | | Canada, Newfoundland (Serusiaux et al. 2011) | | NL2 | ----- | | HQ455096 | ----- |  |
|  | *N. laevigatum* | | Canada, Nova Scotia (Serusiaux et al. 2011) | | NL2 | ----- | | HQ455094 | ----- |  |
|  | *N. laevigatum* | | Finland (Lohtander et al. 2002) | | NL2 | ----- | | AY124143 | ----- |  |
|  | *N. laevigatum* | | France (Lohtander et al. 2002) | | NL2 | ----- | | AY124141 | ----- |  |
|  | *N. laevigatum* | | France, Corsica (Serusiaux et al. 2011) | | NL2 | ----- | | HQ455079 | ----- |  |
|  | *N. laevigatum* | | Norway (Lohtander et al. 2002) | | NL2 | ----- | | AY124142 | ----- |  |
|  | *N. laevigatum* | | Norway (U. Kaasalainen U370) | | NL2 | B1 | | JN857252 | JN857139 |  |
|  | *N. laevigatum* | | Portugal (Serusiaux et al. 2011) | | NL2 | ----- | | HQ455086 | ----- |  |
|  | *N. laevigatum* | | Portugal (Serusiaux et al. 2011) | | NL2 | ----- | | HQ455087 | ----- |  |
|  | *N. laevigatum* | | Portugal (Serusiaux et al. 2011) | | NL2 | ----- | | HQ455088 | ----- |  |
|  | *N. laevigatum* | | Portugal (Serusiaux et al. 2011) | | NL2 | ----- | | HQ455089 | ----- |  |
|  | *N. laevigatum* | | Portugal (Serusiaux et al. 2011) | | NL2 | ----- | | HQ455090 | ----- |  |
|  | *N. laevigatum* | | Portugal (Serusiaux et al. 2011) | | NL2 | ----- | | HQ455091 | ----- |  |
|  | *N. laevigatum* | | Portugal (Serusiaux et al. 2011) | | NL2 | ----- | | HQ455092 | ----- |  |
|  | *N. laevigatum* | | Scotland (Serusiaux et al. 2011) | | NL2 | ----- | | HQ455078 | ----- |  |
|  | *N. laevigatum* | | Spain (Serusiaux et al. 2011) | | NL2 | ----- | | HQ455080 | ----- |  |
|  | *N. laevigatum* | | USA, Maine (Serusiaux et al. 2011) | | NL2 | ----- | | HQ455093 | ----- |  |
|  | *N. laevigatum* | | Canary Islands (Lohtander et al. 2002) | | NL3 | ----- | | AY124140 | ----- |  |
|  | *N. laevigatum* | | Canary Islands (Serusiaux et al. 2011) | | NL3 | ----- | | HQ455098 | ----- |  |
|  | *N. laevigatum* | | Canary Islands (Serusiaux et al. 2011) | | NL4 | ----- | | HQ455077 | ----- |  |
|  | *N. laevigatum* | | Madeira (Lohtander et al. 2002) | | NL5 | ----- | | AY124139 | ----- |  |
|  | *N. laevigatum* | | Madeira (Serusiaux et al. 2011) | | NL5 | ----- | | HQ455097 | ----- |  |
|  | *N. laevigatum* | | Canary Islands (Serusiaux et al. 2011) | | NL6 | ----- | | HQ455081 | ----- |  |
|  | *Nephroma occultum* | | Canada, BC (Piercey-Normore et al. 2006) | | NO1 | ----- | | DQ320560 | ----- |  |
|  | *N. occultum* | | Canada, BC (Piercey-Normore et al. 2006) | | NO2 | ----- | | DQ320559 | ----- |  |
|  | *Nephroma parile* | | Finland (Rikkinen et al. 2002) | | ----- | A1 | | ----- | AF509371 |  |
|  | *N. parile* | | Finland (Rikkinen et al. 2002) | | ----- | A2 | | ----- | AF509389 |  |
|  | *N. parile* | | Finland (Rikkinen et al. 2002) | | ----- | A2 | | ----- | AF509390 |  |
|  | *N. parile* | | Finland (Fedrowitz et al. 2011) | | ----- | A2 | | ----- | HM448620 |  |
|  | *N. parile* | | Finland (Fedrowitz et al. 2011) | | ----- | A2 | | ----- | HM448621 |  |
|  | *N. parile* | | Finland (Fedrowitz et al. 2011) | | ----- | A2 | | ----- | HM448623 |  |
|  | *N. parile* | | Finland (Fedrowitz et al. 2011) | | ----- | A2 | | ----- | HM448624 |  |
|  | *N. parile* | | Finland (Fedrowitz et al. 2011) | | ----- | A2 | | ----- | HM448625 |  |
|  | *N. parile* | | Finland (Fedrowitz et al. 2011) | | ----- | A2 | | ----- | HM448626 |  |
|  | *N. parile* | | Finland (Fedrowitz et al. 2011) | | ----- | A2 | | ----- | HM448627 |  |
|  | *N. parile* | | Finland (Fedrowitz et al. 2011) | | ----- | A2 | | ----- | HM448628 |  |
|  | *N. parile* | | Finland (Fedrowitz et al. 2011) | | ----- | A2 | | ----- | HM448629 |  |
|  | *N. parile* | | Finland (Fedrowitz et al. 2011) | | ----- | A2 | | ----- | HM448630 |  |
|  | *N. parile* | | Finland (Fedrowitz et al. 2011) | | ----- | A2 | | ----- | HM448631 |  |
|  | *N. parile* | | Finland (Fedrowitz et al. 2011) | | ----- | A2 | | ----- | HM448632 |  |
|  | *N. parile* | | Finland (Fedrowitz et al. 2011) | | ----- | A2 | | ----- | HM448633 |  |
|  | *N. parile* | | Finland (Fedrowitz et al. 2011) | | ----- | A2 | | ----- | HM448634 |  |
|  | *N. parile* | | Finland (Fedrowitz et al. 2011) | | ----- | A2 | | ----- | HM448635 |  |
|  | *N. parile* | | Finland (Fedrowitz et al. 2011) | | ----- | A2 | | ----- | HM448636 |  |
|  | *N. parile* | | Finland (Fedrowitz et al. 2011) | | ----- | A2 | | ----- | HM448637 |  |
|  | *N. parile* | | Finland (Fedrowitz et al. 2011) | | ----- | A2 | | ----- | HM448639 |  |
|  | *N. parile* | | Finland (Fedrowitz et al. 2011) | | ----- | A2 | | ----- | HM448641 |  |
|  | *N. parile* | | Finland (Fedrowitz et al. 2011) | | ----- | A2 | | ----- | HM448642 |  |
|  | *N. parile* | | Finland (Fedrowitz et al. 2011) | | ----- | A2 | | ----- | HM448643 |  |
|  | *N. parile* | | Finland (Fedrowitz et al. 2011) | | ----- | A2 | | ----- | HM448644 |  |
|  | *N. parile* | | Finland (Fedrowitz et al. 2011) | | ----- | A2 | | ----- | HM448645 |  |
|  | *N. parile* | | Finland (Fedrowitz et al. 2011) | | ----- | A2 | | ----- | HM448646 |  |
|  | *N. parile* | | Finland (Fedrowitz et al. 2011) | | ----- | A2 | | ----- | HM448647 |  |
|  | *N. parile* | | Finland (Fedrowitz et al. 2011) | | ----- | A2 | | ----- | HM448648 |  |
|  | *N. parile* | | Finland (Fedrowitz et al. 2011) | | ----- | A2 | | ----- | HM448649 |  |
|  | *N. parile* | | Finland (Fedrowitz et al. 2011) | | ----- | A2 | | ----- | HM448650 |  |
|  | *N. parile* | | Finland (Fedrowitz et al. 2011) | | ----- | A2 | | ----- | HM448651 |  |
|  | *N. parile* | | Finland (Fedrowitz et al. 2011) | | ----- | A2 | | ----- | HM448652 |  |
|  | *N. parile* | | Finland (Fedrowitz et al. 2011) | | ----- | A2 | | ----- | HM448653 |  |
|  | *N. parile* | | Finland (Fedrowitz et al. 2011) | | ----- | A2 | | ----- | HM448654 |  |
|  | *N. parile* | | Finland (Fedrowitz et al. 2011) | | ----- | A2 | | ----- | HM448655 |  |
|  | *N. parile* | | Finland (Fedrowitz et al. 2011) | | ----- | A2 | | ----- | HM448658 |  |
|  | *N. parile* | | Finland (Fedrowitz et al. 2011) | | ----- | A2 | | ----- | HM448659 |  |
|  | *N. parile* | | Finland (Fedrowitz et al. 2011) | | ----- | A2 | | ----- | HM448667 |  |
|  | *N. parile* | | Finland (Fedrowitz et al. 2011) | | ----- | A2 | | ----- | HM448668 |  |
|  | *N. parile* | | Finland (Fedrowitz et al. 2011) | | ----- | A2 | | ----- | HM448669 |  |
|  | *N. parile* | | Finland (Fedrowitz et al. 2011) | | ----- | A2 | | ----- | HM448670 |  |
|  | *N. parile* | | Finland (Fedrowitz et al. 2011) | | ----- | A2 | | ----- | HM448673 |  |
|  | *N. parile* | | Finland (Fedrowitz et al. 2011) | | ----- | A2 | | ----- | HM448674 |  |
|  | *N. parile* | | Finland (Rikkinen et al. 2002) | | ----- | A3 | | ----- | AF509392 |  |
|  | *N. parile* | | Finland (Rikkinen et al. 2002) | | ----- | A3 | | ----- | AF509393 |  |
|  | *N. parile* | | Finland (Rikkinen et al. 2002) | | ----- | A3 | | ----- | AF509394 |  |
|  | *N. parile* | | Finland (Fedrowitz et al. 2011) | | ----- | A3 | | ----- | HM448682 |  |
|  | *N. parile* | | Finland (Fedrowitz et al. 2011) | | ----- | A3 | | ----- | HM448683 |  |
|  | *N. parile* | | Finland (Fedrowitz et al. 2011) | | ----- | A3 | | ----- | HM448685 |  |
|  | *N. parile* | | Finland (Fedrowitz et al. 2011) | | ----- | A3 | | ----- | HM448686 |  |
|  | *N. parile* | | Finland (Fedrowitz et al. 2011) | | ----- | A3 | | ----- | HM448687 |  |
|  | *N. parile* | | Finland (Fedrowitz et al. 2011) | | ----- | A3 | | ----- | HM448688 |  |
|  | *N. parile* | | Finland (Fedrowitz et al. 2011) | | ----- | A3 | | ----- | HM448689 |  |
|  | *N. parile* | | Finland (Fedrowitz et al. 2011) | | ----- | A3 | | ----- | HM448690 |  |
|  | *N. parile* | | Finland (Fedrowitz et al. 2011) | | ----- | A3 | | ----- | HM448691 |  |
|  | *N. parile* | | Finland (Lohtander et al. 2002) | | NP1 | ----- | | AY124149 | ----- |  |
|  | *N. parile* | | Finland (Lohtander et al. 2002) | | NP1 | ----- | | AY124150 | ----- |  |
|  | *N. parile* | | Finland (Lohtander et al. 2002) | | NP1 | ----- | | AY124151 | ----- |  |
|  | *N. parile* | | Finland (Fedrowitz et al. 2011) | | NP1 | ----- | | HM448781 | ----- |  |
|  | *N. parile* | | Greenland (Lohtander et al. 2002) | | NP1 | ----- | | AY124147 | ----- |  |
|  | *N. parile* | | Japan (G. Thor (GT 23856) (KU492a)) | | NP1 | A1 | | JN857253 | JN857140 |  |
|  | *N. parile* | | Finland (Fedrowitz et al. 2011) | | NP1 | A2 | | HM448776 | HM448616 |  |
|  | *N. parile* | | Finland (Fedrowitz et al. 2011) | | NP1 | A2 | | HM448773 | HM448617 |  |
|  | *N. parile* | | Finland (Fedrowitz et al. 2011) | | NP1 | A2 | | HM448777 | HM448618 |  |
|  | *N. parile* | | Finland (Fedrowitz et al. 2011) | | NP1 | A2 | | HM448772 | HM448619 |  |
|  | *N. parile* | | Finland (Fedrowitz et al. 2011) | | NP1 | A2 | | HM448779 | HM448622 |  |
|  | *N. parile* | | Finland (Fedrowitz et al. 2011) | | NP1 | A2 | | HM448782 | HM448638 |  |
|  | *N. parile* | | Finland (Fedrowitz et al. 2011) | | NP1 | A2 | | HM448771 | HM448640 |  |
|  | *N. parile* | | Finland (Fedrowitz et al. 2011) | | NP1 | A2 | | HM448780 | HM448660 |  |
|  | *N. parile* | | Finland (Fedrowitz et al. 2011) | | NP1 | A2 | | HM448778 | HM448675 |  |
|  | *N. parile* | | Finland (Fedrowitz et al. 2011) | | NP1 | A2 | | HM448774 | HM448676 |  |
|  | *N. parile* | | Finland (Fedrowitz et al. 2011) | | NP1 | A2 | | HM448775 | HM448677 |  |
|  | *N. parile* | | Canada QC (H. Coffey & C. Freebury KU1003) | | NP1 | A3 | | JN857254 | JN857141 |  |
|  | *N. parile* | | Finland (Fedrowitz et al. 2011) | | NP1 | A3 | | HM448785 | HM448678 |  |
|  | *N. parile* | | Finland (Fedrowitz et al. 2011) | | NP1 | A3 | | HM448786 | HM448679 |  |
|  | *N. parile* | | Finland (Fedrowitz et al. 2011) | | NP1 | A3 | | HM448783 | HM448680 |  |
|  | *N. parile* | | Finland (Fedrowitz et al. 2011) | | NP1 | A3 | | HM448787 | HM448681 |  |
|  | *N. parile* | | Finland (Fedrowitz et al. 2011) | | NP1 | A3 | | HM448784 | HM448684 |  |
|  | *N. parile* | | Finland (J. Rikkinen (JR08J2A) (KUJ46)) | | NP1 | A3 | | JN857255 | JN857142 |  |
|  | *N. parile* | | Finland (J. Rikkinen (JR08S1O) (KUJ114)) | | NP1 | A3 | | JN857256 | JN857143 |  |
|  | *N. parile* | | Finland (J. Rikkinen (JR08S1Q) (KUJ116)) | | NP1 | A3 | | JN857257 | JN857144 |  |
|  | *N. parile* | | Finland (J. Rikkinen (JR08V1I) (KUJ138)) | | NP1 | A3 | | JN857258 | JN857145 |  |
|  | *N. parile* | | Finland (J. Rikkinen (JR201008) (KU515)) | | NP1 | A3 | | JN857259 | JN857146 |  |
|  | *N. parile* | | Finland (J. Rikkinen U562b) | | NP1 | A3 | | JN857260 | JN857147 |  |
|  | *N. parile* | | Norway (U. Kaasalainen U371) | | NP1 | A3 | | JN857261 | JN857148 |  |
|  | *N. parile* | | Norway (U. Kaasalainen U387) | | NP1 | A3 | | JN857262 | JN857149 |  |
|  | *N. parile* | | Scotland (K. Fedrowitz (KUS3)) | | NP1 | A3 | | JN857263 | JN857150 |  |
|  | *N. parile* | | Scotland (K. Fedrowitz (KUS36A)) | | NP1 | A3 | | JN857264 | JN857151 |  |
|  | *N. parile* | | Scotland (K. Fedrowitz (KUS36B)) | | NP1 | A3 | | JN857265 | JN857152 |  |
|  | *N. parile* | | Scotland (K. Fedrowitz (KUS36C)) | | NP1 | A3 | | JN857266 | JN857153 |  |
|  | *N. parile* | | Scotland (K. Fedrowitz (KUS40)) | | NP1 | A3 | | JN857267 | JN857154 |  |
|  | *N. parile* | | Scotland (K. Fedrowitz KUS44) | | NP1 | A3 | | JN857268 | JN857155 |  |
|  | *N. parile* | | Scotland (K. Fedrowitz KUS53) | | NP1 | A3 | | JN857269 | JN857156 |  |
|  | *N. parile* | | Sweden (A. Frisch (AF1/npa) (KU501)) | | NP1 | A3 | | JN857270 | JN857157 |  |
|  | *N. parile* | | Sweden (A. Frisch (AF2/npa) (KU502)) | | NP1 | A3 | | JN857271 | JN857158 |  |
|  | *N. parile* | | Sweden (K. Fedrowitz KU497) | | NP1 | A3 | | JN857272 | JN857159 |  |
|  | *N. parile* | | Sweden (K. Fedrowitz KU498) | | NP1 | A3 | | JN857273 | JN857160 |  |
|  | *N. parile* | | Sweden (K. Fedrowitz KU500) | | NP1 | A3 | | JN857274 | JN857161 |  |
|  | *N. parile* | | Finland (J. Rikkinen (JR08J4D) (KUJ64)) | | NP2 | A3 | | JN857275 | JN857162 |  |
|  | *N. parile* | | Finland (J. Rikkinen (JR08S1P) (KUJ115)) | | NP3 | A3 | | JN857276 | JN857163 |  |
|  | *N. parile* | | Japan (A. Frisch (10/Jp3) (KU523b)) | | NP4 | A1 | | JN857277 | JN857164 |  |
|  | *N. parile* | | Japan (A. Frisch (10/Jp5) (KU525b)) | | NP4 | A1 | | JN857278 | JN857165 |  |
|  | *N. parile* | | Japan (G. Thor (GT 24040) (KU494a)) | | NP4 | A1 | | JN857279 | JN857166 |  |
|  | *N. parile* | | Canada SW (Lohtander et al. 2002) | | NP5 | ----- | | AY124145 | ----- |  |
|  | *N. parile* | | Canada, BC (Serusiaux et al. 2011) | | NP5 | ----- | | HQ455102 | ----- |  |
|  | *N. parile* | | USA, Montana (Serusiaux et al. 2011) | | NP5 | ----- | | HQ455099 | ----- |  |
|  | *N. parile* | | USA, Montana (Serusiaux et al. 2011) | | NP5 | ----- | | HQ455100 | ----- |  |
|  | *N. parile* | | Canada E (Lohtander et al. 2002) | | NP6 | ----- | | AY124148 | ----- |  |
|  | *N. parile* | | Canada, BC (Serusiaux et al. 2011) | | NP6 | ----- | | HQ455101 | ----- |  |
|  | *N. parile* | | Canada, BC (Serusiaux et al. 2011) | | NP6 | ----- | | HQ455103 | ----- |  |
|  | *N. parile* | | Norway (U. Kaasalainen U372) | | NP6 | A3 | | JN857280 | JN857167 |  |
|  | *N. parile* | | South Korea (Unpublished) | | NP6 | ----- | | DQ066708 | ----- |  |
|  | *N. parile* | | Sweden (K. Fedrowitz KU499) | | NP6 | A3 | | JN857281 | JN857168 |  |
|  | *N. parile* | | Finland (J. Rikkinen (JR08S1R) (KUJ117)) | | NP7 | A7 | | JN857282 | JN857169 |  |
|  | *N. parile* | | Canada QC (H. Coffey & C. Freebury KU1001) | | NP8 | F1 | | JN857283 | JN857170 |  |
|  | *Nephroma plumbeum* | | New Zealand (?) (Unpublished) | | Nephroma plumbeum | ----- | | AF350288 | ----- |  |
|  | *Nephroma resupinatum* | | Finland (Fedrowitz et al. 2011) | | ----- | A1 | | ----- | HM448565 |  |
|  | *N. resupinatum* | | Finland (Fedrowitz et al. 2011) | | ----- | A1 | | ----- | HM448567 |  |
|  | *N. resupinatum* | | Finland (Fedrowitz et al. 2011) | | ----- | A1 | | ----- | HM448568 |  |
|  | *N. resupinatum* | | Finland (Fedrowitz et al. 2011) | | ----- | A1 | | ----- | HM448569 |  |
|  | *N. resupinatum* | | Finland (Fedrowitz et al. 2011) | | ----- | A1 | | ----- | HM448570 |  |
|  | *N. resupinatum* | | Finland (Fedrowitz et al. 2011) | | ----- | A1 | | ----- | HM448571 |  |
|  | *N. resupinatum* | | Finland (Fedrowitz et al. 2011) | | ----- | A1 | | ----- | HM448573 |  |
|  | *N. resupinatum* | | Finland (Fedrowitz et al. 2011) | | ----- | A1 | | ----- | HM448574 |  |
|  | *N. resupinatum* | | Finland (Fedrowitz et al. 2011) | | ----- | A1 | | ----- | HM448575 |  |
|  | *N. resupinatum* | | Finland (Fedrowitz et al. 2011) | | ----- | A1 | | ----- | HM448577 |  |
|  | *N. resupinatum* | | Finland (Fedrowitz et al. 2011) | | ----- | A1 | | ----- | HM448578 |  |
|  | *N. resupinatum* | | Finland (Fedrowitz et al. 2011) | | ----- | A1 | | ----- | HM448580 |  |
|  | *N. resupinatum* | | Finland (Fedrowitz et al. 2011) | | ----- | A1 | | ----- | HM448581 |  |
|  | *N. resupinatum* | | Finland (Fedrowitz et al. 2011) | | ----- | A1 | | ----- | HM448582 |  |
|  | *N. resupinatum* | | Finland (Fedrowitz et al. 2011) | | ----- | A1 | | ----- | HM448584 |  |
|  | *N. resupinatum* | | Finland (Fedrowitz et al. 2011) | | ----- | A1 | | ----- | HM448585 |  |
|  | *N. resupinatum* | | Finland (Fedrowitz et al. 2011) | | ----- | A1 | | ----- | HM448587 |  |
|  | *N. resupinatum* | | Finland (Fedrowitz et al. 2011) | | ----- | A1 | | ----- | HM448588 |  |
|  | *N. resupinatum* | | Finland (Fedrowitz et al. 2011) | | ----- | A1 | | ----- | HM448589 |  |
|  | *N. resupinatum* | | Finland (Fedrowitz et al. 2011) | | ----- | A1 | | ----- | HM448590 |  |
|  | *N. resupinatum* | | Finland (Fedrowitz et al. 2011) | | ----- | A1 | | ----- | HM448594 |  |
|  | *N. resupinatum* | | Finland (Fedrowitz et al. 2011) | | ----- | A1 | | ----- | HM448596 |  |
|  | *N. resupinatum* | | Finland (Fedrowitz et al. 2011) | | ----- | A1 | | ----- | HM448598 |  |
|  | *N. resupinatum* | | Finland (Fedrowitz et al. 2011) | | ----- | A1 | | ----- | HM448600 |  |
|  | *N. resupinatum* | | Finland (Fedrowitz et al. 2011) | | ----- | A1 | | ----- | HM448601 |  |
|  | *N. resupinatum* | | Finland (Fedrowitz et al. 2011) | | ----- | A1 | | ----- | HM448602 |  |
|  | *N. resupinatum* | | Finland (Fedrowitz et al. 2011) | | ----- | A1 | | ----- | HM448603 |  |
|  | *N. resupinatum* | | Finland (Fedrowitz et al. 2011) | | ----- | A1 | | ----- | HM448604 |  |
|  | *N. resupinatum* | | Finland (Fedrowitz et al. 2011) | | ----- | A1 | | ----- | HM448605 |  |
|  | *N. resupinatum* | | Finland (Fedrowitz et al. 2011) | | ----- | A1 | | ----- | HM448606 |  |
|  | *N. resupinatum* | | Finland (Rikkinen et al. 2002) | | ----- | A1 | | ----- | AF509372 |  |
|  | *N. resupinatum* | | Finland (Rikkinen et al. 2002) | | ----- | A1 | | ----- | AF509373 |  |
|  | *N. resupinatum* | | Finland (Rikkinen et al. 2002) | | ----- | A1 | | ----- | AF509374 |  |
|  | *N. resupinatum* | | Finland (Rikkinen et al. 2002) | | ----- | A1 | | ----- | AF509375 |  |
|  | *N. resupinatum* | | Finland (Rikkinen et al. 2002) | | ----- | A1 | | ----- | AF509376 |  |
|  | *N. resupinatum* | | Finland (Rikkinen et al. 2002) | | ----- | A3 | | ----- | AF509395 |  |
|  | *N. resupinatum* | | Finland (Fedrowitz et al. 2011) | | ----- | A4 | | ----- | HM448702 |  |
|  | *N. resupinatum* | | Finland (Fedrowitz et al. 2011) | | ----- | A4 | | ----- | HM448703 |  |
|  | *N. resupinatum* | | Finland (Fedrowitz et al. 2011) | | ----- | A4 | | ----- | HM448704 |  |
|  | *N. resupinatum* | | Finland (Fedrowitz et al. 2011) | | ----- | A4 | | ----- | HM448705 |  |
|  | *N. resupinatum* | | Finland (Fedrowitz et al. 2011) | | ----- | A4 | | ----- | HM448706 |  |
|  | *N. resupinatum* | | Finland (Fedrowitz et al. 2011) | | ----- | A4 | | ----- | HM448707 |  |
|  | *N. resupinatum* | | Finland (Fedrowitz et al. 2011) | | ----- | A4 | | ----- | HM448708 |  |
|  | *N. resupinatum* | | Finland (Fedrowitz et al. 2011) | | ----- | A4 | | ----- | HM448710 |  |
|  | *N. resupinatum* | | Finland (Fedrowitz et al. 2011) | | ----- | A4 | | ----- | HM448711 |  |
|  | *N. resupinatum* | | Finland (Fedrowitz et al. 2011) | | ----- | A4 | | ----- | HM448714 |  |
|  | *N. resupinatum* | | Finland (Fedrowitz et al. 2011) | | ----- | A4 | | ----- | HM448716 |  |
|  | *N. resupinatum* | | Finland (Fedrowitz et al. 2011) | | ----- | A4 | | ----- | HM448717 |  |
|  | *N. resupinatum* | | Finland (Fedrowitz et al. 2011) | | ----- | A4 | | ----- | HM448718 |  |
|  | *N. resupinatum* | | Finland (Fedrowitz et al. 2011) | | ----- | A4 | | ----- | HM448721 |  |
|  | *N. resupinatum* | | Finland (Fedrowitz et al. 2011) | | ----- | B1 | | ----- | HM448694 |  |
|  | *N. resupinatum* | | Finland (Paulsrud et al. 1998) | | ----- | B1 | | ----- | AF055660 |  |
|  | *N. resupinatum* | | Finland (Rikkinen et al. 2002) | | ----- | B1 | | ----- | AF509397 |  |
|  | *N. resupinatum* | | Finland (Rikkinen et al. 2002) | | ----- | B1 | | ----- | AF509398 |  |
|  | *N. resupinatum* | | USA, Oregon (Paulsrud et al. 2000) | | ----- | C2 | | ----- | AF176595 |  |
|  | *N. resupinatum* | | USA, Oregon (Rikkinen et al. 2002) | | ----- | A12 | | ----- | AF509405 |  |
|  | *N. resupinatum* | | Finland (U. Kaasalainen U563b) | | NR1 | A2 | | JN857284 | JN857171 |  |
|  | *N. resupinatum* | | Finland (Lohtander et al. 2002) | | NR2 | ----- | | AY124104 | ----- |  |
|  | *N. resupinatum* | | Sweden (A. Frisch (AF2/nre) (KU509)) | | NR2 | A3 | | JN857285 | JN857172 |  |
|  | *N. resupinatum* | | Finland (Fedrowitz et al. 2011) | | NR3 | A1 | | HM448762 | HM448599 |  |
|  | *N. resupinatum* | | Finland (Lohtander et al. 2002) | | NR3 | ----- | | AY124103 | ----- |  |
|  | *N. resupinatum* | | Sweden (A. Frisch (AF1/nre) (KU508)) | | NR3 | A3 | | JN857286 | JN857173 |  |
|  | *N. resupinatum* | | Finland (Fedrowitz et al. 2011) | | NR4 | A1 | | HM448759 | HM448564 |  |
|  | *N. resupinatum* | | Finland (Fedrowitz et al. 2011) | | NR4 | A1 | | HM448760 | HM448566 |  |
|  | *N. resupinatum* | | Finland (Fedrowitz et al. 2011) | | NR4 | A1 | | HM448765 | HM448572 |  |
|  | *N. resupinatum* | | Finland (Fedrowitz et al. 2011) | | NR4 | A1 | | HM448764 | HM448576 |  |
|  | *N. resupinatum* | | Finland (Fedrowitz et al. 2011) | | NR4 | A1 | | HM448758 | HM448579 |  |
|  | *N. resupinatum* | | Finland (Fedrowitz et al. 2011) | | NR4 | A1 | | HM448763 | HM448583 |  |
|  | *N. resupinatum* | | Finland (Fedrowitz et al. 2011) | | NR4 | A1 | | HM448766 | HM448586 |  |
|  | *N. resupinatum* | | Finland (Fedrowitz et al. 2011) | | NR4 | A1 | | HM448761 | HM448591 |  |
|  | *N. resupinatum* | | Finland (Fedrowitz et al. 2011) | | NR4 | A4 | | HM448769 | HM448709 |  |
|  | *N. resupinatum* | | Finland (Fedrowitz et al. 2011) | | NR4 | A4 | | HM448770 | HM448720 |  |
|  | *N. resupinatum* | | Finland (Fedrowitz et al. 2011) | | NR4 | B1 | | HM448767 | HM448692 |  |
|  | *N. resupinatum* | | Finland (Fedrowitz et al. 2011) | | NR4 | B1 | | HM448768 | HM448695 |  |
|  | *N. resupinatum* | | Finland (Lohtander et al. 2002) | | NR4 | ----- | | AY124097 | ----- |  |
|  | *N. resupinatum* | | Finland (Lohtander et al. 2002) | | NR4 | ----- | | AY124100 | ----- |  |
|  | *N. resupinatum* | | Finland (Lohtander et al. 2002) | | NR4 | ----- | | AY124101 | ----- |  |
|  | *N. resupinatum* | | Finland (Lohtander et al. 2002) | | NR4 | ----- | | AY124102 | ----- |  |
|  | *N. resupinatum* | | USA, SW (Lohtander et al. 2002) | | NR4 | ----- | | AY124096 | ----- |  |
|  | *N. resupinatum* | | Japan (G. Thor (GT 24097) (KU495)) | | NR5 | A1 | | JN857287 | JN857174 |  |
|  | *N. resupinatum* | | Japan (G. Thor (GT 24175) (KU496a)) | | NR5 | ----- | | JN857288 | JN857175 |  |
|  | *N. resupinatum* | | Japan (A. Frisch (10/Jp2) (KU522a)) | | NR6 | A6 | | JN857289 | JN857176 |  |
|  | *N. resupinatum* | | South Korea (Unpublished) | | NR6 | ----- | | DQ066709 | ----- |  |
|  | *N. resupinatum* | | South Korea (Unpublished) | | NR6 | ----- | | DQ066710 | ----- |  |
|  | *N. resupinatum* | | USA, California (U. Kaasalainen U181) | | NR7 | A5 | | JN857290 | JN857177 |  |
|  | *N. resupinatum* | | USA, California (U. Kaasalainen U188) | | NR8 | B3 | | JN857291 | JN857178 |  |
|  | *N. resupinatum* | | USA, Oregon (Lohtander et al. 2002) | | NR9 | ----- | | AY124099 | ----- |  |
|  | *N. resupinatum* | | USA, Oregon (Lohtander et al. 2002) | | NR10 | ----- | | AY124098 | ----- |  |
|  | *Nephroma rufum* | | New Zealand (?) (Unpublished) | | Nephroma rufum | ----- | | AF350289 | ----- |  |
|  | *Nephroma squamigerum* | | Japan (A. Frisch (10/Jp12) (KU526)) | | NS1 | A6 | | JN857292 | JN857179 |  |
|  | *N. squamigerum* | | Japan (A. Frisch (10/Jp4) (KU524a)) | | NS2 | A2 | | JN857293 | JN857180 |  |
|  | *N. squamigerum* | | Japan (G. Thor (GT 23855) (KU517a)) | | NS3 | A2 | | JN857294 | JN857181 |  |
|  | *N. squamigerum* | | Japan (G. Thor (600A2/3) (KU556)) | | NS4 | A1 | | JN857295 | JN857182 |  |
|  | *N. squamigerum* | | Japan (A. Frisch (10/Jp1) (KU521a)) | | NS4 | A2 | | JN857296 | JN857183 |  |
|  | *N. squamigerum* | | Japan (G. Thor (GT 24506) (KU516)) | | NS4 | A2 | | JN857297 | JN857184 |  |
|  | *N. squamigerum* | | South Korea (Unpublished) | | NS5 | ----- | | DQ066701 | ----- |  |
|  | *N. squamigerum* | | South Korea (Unpublished) | | NS6 | ----- | | DQ066700 | ----- |  |
|  | *Nephroma sulcatum* | | Canary Islands (Serusiaux et al. 2011) | | NSU1 | ----- | | HQ455105 | ----- |  |
|  | *N. sulcatum* | | Canary Islands (Serusiaux et al. 2011) | | NSU1 | ----- | | HQ455104 | ----- |  |
|  | *N. sulcatum* | | Canary Islands (Lohtander et al. 2002) | | NSU2 | ----- | | AY124146 | ----- |  |
|  | *Nephroma tangeriense* | | Canary Islands (J. Rikkinen (544) (KU544)) | | NT1 | C1 | | JN857298 | JN857185 |  |
|  | *N. tangeriense* | | Canary Islands (J. Rikkinen (JR012001) (KU540)) | | NT1 | C1 | | JN857299 | JN857186 |  |
|  | *N. tangeriense* | | Canary Islands (J. Rikkinen (JR012002) (KU541)) | | NT1 | C1 | | JN857300 | JN857187 |  |
|  | *N. tangeriense* | | Canary Islands (J. Rikkinen (JR012003) (KU542)) | | NT1 | C1 | | JN857301 | JN857188 |  |
|  | *N. tangeriense* | | Canary Islands (J. Rikkinen (JR012006) (KU545)) | | NT1 | C1 | | JN857302 | JN857189 |  |
|  | *N. tangeriense* | | Canary Islands (J. Rikkinen (JR012007) (KU546)) | | NT1 | C1 | | JN857303 | JN857190 |  |
|  | *N. tangeriense* | | Canary Islands (J. Rikkinen (JR012008) (KU547)) | | NT1 | C1 | | JN857304 | JN857191 |  |
|  | *N. tangeriense* | | Canary Islands (J. Rikkinen (JR012004) (KU543a)) | | NT1 | C4 | | JN857305 | JN857192 |  |
|  | *N. tangeriense* | | Canary Islands (Lohtander et al. 2002) | | NT1 | ----- | | AY124152 | ----- |  |
|  | *N. tangeriense* | | Portugal (Lohtander et al. 2002) | | NT1 | ----- | | AY124153 | ----- |  |
|  | *N. tangeriense* | | Spain (Lohtander et al. 2002) | | NT1 | ----- | | AY124154 | ----- |  |
|  | *N. tangeriense* | | Spain (Serusiaux et al. 2011) | | NT1 | ----- | | HQ455106 | ----- |  |
|  | *N. tangeriense* | | Spain (Serusiaux et al. 2011) | | NT2 | ----- | | HQ455107 | ----- |  |
|  | *Nephroma washingtoniense* | | USA, California (U. Kaasalainen U187) | | NW1 | B3 | | JN857306 | JN857193 |  |
|  | *N. washingtoniense* | | USA, Oregon (U. Kaasalainen U143) | | NW2 | C3 | | JN857307 | JN857194 |  |
|  | *N. washingtoniense* | | USA, Oregon (Lohtander et al. 2002) | | NW2 | ----- | | AY124144 | ----- |  |
|  | *N. washingtoniense* | | USA, Oregon (Serusiaux et al. 2011) | | NW2 | ----- | | HQ455076 | ----- |  |
|  | *N. washingtoniense* | | USA, Oregon (U. Kaasalainen U146) | | NW3 | C3 | | JN857308 | JN857195 |  |
|  | *Nephroma venosum* | | Azores (Serusiaux et al. 2011) | | NV1 | ----- | | HQ455112 | ----- |  |
|  | *N. venosum* | | Azores (Serusiaux et al. 2011) | | NV2 | ----- | | HQ455108 | ----- |  |
|  | *N. venosum* | | Azores (Serusiaux et al. 2011) | | NV2 | ----- | | HQ455109 | ----- |  |
|  | *N. venosum* | | Azores (Serusiaux et al. 2011) | | NV2 | ----- | | HQ455110 | ----- |  |
|  | *N. venosum* | | Azores (Serusiaux et al. 2011) | | NV3 | ----- | | HQ455111 | ----- |  |
|  | *Sticta limbata* | | USA, California (U. Kaasalainen U191) | | Sticta limbata | ----- | | JN857309 | JN857196 |  |
